# Effects of 1p/19q Codeletion on Immune Phenotype in Low Grade Glioma

**DOI:** 10.3389/fncel.2021.704344

**Published:** 2021-07-16

**Authors:** Lei Lv, Yuliu Zhang, Yujia Zhao, Qinqin Wei, Ye Zhao, Qiyi Yi

**Affiliations:** ^1^Anhui Cancer Hospital, West Branch of the First Affiliated Hospital of USTC, Division of Life Sciences and Medicine, University of Science and Technology of China, Hefei, China; ^2^Department of Thoracic Surgery, Dingyuan County General Hospital of Chuzhou City in Anhui, Anhui, China; ^3^School of Basic Medical Sciences, Anhui Medical University, Hefei, China

**Keywords:** low-grade glioma, immune checkpoint genes, tumor immune cell infiltration, tumor immune microenvironment, IDH mutation, 1p/19q codeletion

## Abstract

**Background:** Chromosome 1p/19q codeletion is one of the most important genetic alterations for low grade gliomas (LGGs), and patients with 1p/19q codeletion have significantly prolonged survival compared to those without the codeletion. And the tumor immune microenvironment also plays a vital role in the tumor progression and prognosis. However, the effect of 1p/19q codeletion on the tumor immune microenvironment in LGGs is unclear.

**Methods:** Immune cell infiltration of 281 LGGs from The Cancer Genome Atlas (TCGA) and 543 LGGs from the Chinese Glioma Genome Atlas (CGGA) were analyzed for immune cell infiltration through three bioinformatics tools: ESTIMATE algorithm, TIMER, and xCell. The infiltrating level of immune cells and expression of immune checkpoint genes were compared between different groups classified by 1p/19q codeletion and IDH (isocitrate dehydrogenase) mutation status. The differential biological processes and signaling pathways were evaluated through Gene Set Enrichment Analysis (GSEA). Correlations were analyzed using Spearman correlation.

**Results:** 1p/19q codeletion was associated with immune-related biological processes in LGGs. The infiltrating level of multiple kinds of immune cells and expression of immune checkpoint genes were significantly lower in 1p/19q codeletion LGGs compared to 1p/19q non-codeletion cohorts. There are 127 immune-related genes on chromosome 1p or 19q, such as TGFB1, JAK1, and CSF1. The mRNA expression of these genes was positively correlated with their DNA copy number. These genes are distributed in multiple immune categories, such as chemokines/cytokines, TGF-β family members, and TNF family members, regulating immune cell infiltration and expression of the immune checkpoint genes in tumors.

**Conclusion:** Our results indicated that 1p/19q codeletion status is closely associated with the immunosuppressive microenvironment in LGGs. LGGs with 1p/19q codeletion display less immune cell infiltration and lower expression of immune checkpoint genes than 1p/19q non-codeletion cases. Mechanistically, this may be, at least in part, due to the deletion of copy number of immune-related genes in LGGs with 1p/19q codeletion. Our findings may be relevant to investigate immune evasion in LGGs and contribute to the design of immunotherapeutic strategies for patients with LGGs.

## Introduction

Gliomas are central nervous system tumors arising from glioma stem cells (GSCs) that comprise approximately 30% of all primary brain and CNS tumors (Ma et al., 2018; Ostrom et al., 2019), which are categorized into low grade glioma (LGG) and high grade glioma (glioblastoma multiforme, GBM). LGG includes diffuse low-grade and intermediate-grade gliomas (WHO grades II and III, respectively) (Louis et al., 2016). Although LGG patients have better survival than GBM patients, all LGGs have malignant potential to grow invasively and progress to GBMs. The research into glioma in the past decades has led to the discovery of numerous genetic/epigenetic alterations in LGGs. Two of these alterations are particularly noteworthy, as they occur early during glioma formation (Jenkins et al., 2006), are prevalent and strongly associated with overall survival (OS) and progression free survival (PFS) in glioma (Weller et al., 2012). The first alteration to be identified was the codeletion of both the short arm of chromosome 1 (1p) and the long arm of chromosome 19 (19q) (1p/19q codeletion). The second was a mutation in either IDH1 or IDH2 (IDH mutation). In the 2016 WHO Classification of CNS (Central Nervous System) tumors, IDH mutation and 1p/19q codeletion were combined with the traditional histopathological examination to form an integrated diagnosis of LGG. As nearly all 1p/19q co-deleted LGGs have IDH1 or IDH2 mutations (Labussiere et al., 2010), LGGs can be assigned into three subgroups: (i) IDH mutation and 1p/19q codeletion (muIDH + Codel); (ii) IDH mutation and no 1p/19q codeletion (muIDH + Non-codel), and (iii) IDH wild-type and no 1p/19q codeletion (wtIDH + Non-codel). Among these subgroups, the “muIDH + Codel” subgroup has the best prognosis, and 1p/19q co-deletion is an independent favorable prognostic factor on overall survival as reported by many studies (Kujas et al., 2005; Jenkins et al., 2006; Tews et al., 2006; Iwamoto et al., 2008), but the underlying mechanism is still unclear. Previous reports showed that genes located on chromosome arms 1p and 19q (1p/19q genes) were differentially expressed between 1p/19q codel and non-codel gliomas (Tews et al., 2006), suggesting that at least some of these genes may contribute to the better survival of patients with 1p/19q co-deleted gliomas. On the other hand, mutated IDH1/2 could produce (R)-2-hydroxyglutarate (2HG). And the accumulation of 2HG could inhibit key histone demethylase enzymes, leading to DNA hypermethylation phenotype and the repression of multiple genes (Dang et al., 2009; Turcan et al., 2012).

Multiple factors contribute to aggressiveness in solid tumors, one of which is tumor-infiltrating immune cells (TIICs). The level of specific TIICs affects tumor immune evasion and patient survival, and significant infiltration of immune cells is linked to poor prognosis in many types of cancer (Kitamura et al., 2015; Bense et al., 2017; Zhou et al., 2019). Although the brain has been regarded as an immune-privileged organ owing to the blood-brain barrier (BBB) and the lack of a conventional lymphatic drainage system, glioma cells secrete numerous chemokines, cytokines, and growth factors that promote the infiltration of various immune cells such as peripheral macrophages, leukocytes and CD4^+^ T cells into the tumor (Badie and Schartner, 2000; Fecci et al., 2006; Domingues et al., 2016). In recent years, several studies have shown that IDH mutation could repress the infiltration of immune cells and mediate immune evasion in gliomas (Amankulor et al., 2017; Berghoff et al., 2017; Kohanbash et al., 2017; Bunse et al., 2018; Richardson et al., 2019). However, whether and how 1p/19q codel affects infiltration of immune cells and contributes to immune evasion remains unclear. In addition, 1p/19q codeletion and IDH mutation are closely associated. Nearly all 1p/19q codeleted gliomas have IDH1/2 mutations (Labussiere et al., 2010). Thus, ignoring the effect of 1p/19q codeletion while estimating the effect of IDH mutation on immune infiltration, or vice versa, may lead to serious bias.

In this study, we compared the Biological Processes (BPs) between 1p/19q codeleted and non-codeleted LGGs using Gene Set Enrichment Analysis (GSEA). We found that 1p/19q codeleted LGGs had significantly lower activity of immune-related BPs compared to non-codeleted tumors. Further analysis showed that 1p/19q codeleted LGGs had lower levels of TIICs and decreased expression levels of multiple immune checkpoint genes, such as PD1 and its ligands PD-L1/2, than non-codeleted cases. Mechanically, immune-related signaling pathways were impaired due to the loss of hundreds of immune-related genes, which were located on chromosome 1p or 19q in 1p/19q codeleted LGGs.

## Materials and Methods

### Sample and Data Collection

All data analyzed in this study are publicly and freely available. Data from three publicly available datasets were collected from online sources. Copy number (gene-level), gene expression (RNA-seq), and clinical information (including overall survival) of TCGA LGG were obtained from the UCSC Xena Browser^1^. Molecular classifications (including IDH mutation and 1p/19q codel status) of TCGA LGG were obtained from supplementary material from a paper authored by “The Cancer Genome Atlas Research Network”^2^ (Cancer Genome Atlas Research Network et al., 2015). Gene expression (RNA-seq) and clinical information (including overall survival, IDH mutation, and 1p/19q codel status) of CGGA mRNAseq_325 and mRNAseq_693 cohorts, which were last updated on 11/28/2019, were download from the website of Chinese Glioma Genome Atlas (CGGA)^3^.

### Gene Set Enrichment Analysis (GSEA)

Log2FC values of each gene generated using Limma package in R by analyzing differentially expressed genes between two groups of LGGs were enrolled to create the pre-ranked gene list. Then, a pre-ranked GSEA analysis was performed on GO biological process (BP) database (c5.bp.v7.0.symbols.gmt) or the Hallmark signature database (h.all.v7.0.symbols.gmt) of Molecular Signatures Database (MSigDB) with the pre-ranked list (Subramanian et al., 2005).

### Estimation of Infiltrating Level of Immune Cells

R package ESTIMATE was used to evaluate the overall infiltrating level of stromal and immune cells through “stromal score” and “immune score” and infer the tumor purity through “ESTIMATE score” (Yoshihara et al., 2013). Tumor Immune Estimation Resource (TIMER) was used to estimate the infiltrating level of six immune cell types: B cells, CD4^+^ T cells, CD8^+^ T cells, neutrophils, macrophages, and dendritic cells in LGGs based on a list of immune-specific markers and immune cell expression signatures^4^. The outcome of this method has been validated using pathological estimations (Yoshihara et al., 2013). xCell, a method based on ssGSEA that estimating the abundance scores of 64 kinds of cells, was used to evaluate the proportion of the 34 types of immune cell: B-cells, Memory B-cells, naive B-cells, pro B-cells, Class-switched memory B-cells, CD4^+^ T-cells, CD4^+^ memory T-cells, CD4^+^ naive T-cells, CD8^+^ naive T-cells, CD8^+^ T-cells, CD4^+^ Tcm, CD4^+^ Tem, CD8^+^ Tcm, CD8^+^ Tem, NK cells, NKT, Plasma cells, Tgd cells, Th1 cells, Th2 cells, Tregs, Dendritic cells, Activated dendritic cells, Immature dendritic cells, Conventional dendritic cells, Plasmacytoid dendritic cells, Macrophages, Macrophages M1, Macrophages M2, Basophils, Eosinophils, Mast cells, Monocytes, Neutrophils^5^ (Aran et al., 2017).

### Immune-Related Genes

The immunology database and analysis portal (ImmPort) system is an archival repository and dissemination vehicle for clinical and molecular datasets created by research consortia funded by the National Institute of Allergy and Infectious Diseases Division of Allergy, Immunology, and Transplantation (Bhattacharya et al., 2018). Immune-related genes list file “Geneappend3.xls” was downloaded from Immport^6^. And the genomic location of each gene was inquired through HGNC (HUGO Gene Nomenclature Committee)^7^. The list of genes utilized as specific markers of the indicated immune cell types was obtained from Table S4d of Davoli et al. (2017).

### Statistical Analysis

Differences between the two groups were evaluated using the Student’s *t*-test for normally distributed data or Mann-Whitney test for non-normally distributed data. Normality of data was determined by Kolmogorov-Smirnov test. *F*-test was used to compare the variance of two samples before Student’s *t*-test. Welch’s correction is applied to Student’s *t*-test if the null hypothesis of equality of variances is rejected. Correlation analysis was performed using the Spearman’s test. Kaplan-Meier analysis using the “survival” and “survminer” package was performed to compare the patients’ survival time between the two groups. Statistical analysis was performed with GraphPad Prism 8 (GraphPad Software, San Diego, CA, USA) or packages in R software. For the analysis, *P* value < 0.05 was considered statistically significant. ^∗^*p* < 0.05, ^∗∗^*p* < 0.01, ^∗∗∗^*p* < 0.001, ^****^p < 0.0001.

## Results

### Patient Characteristics

**FIGURE 1:**
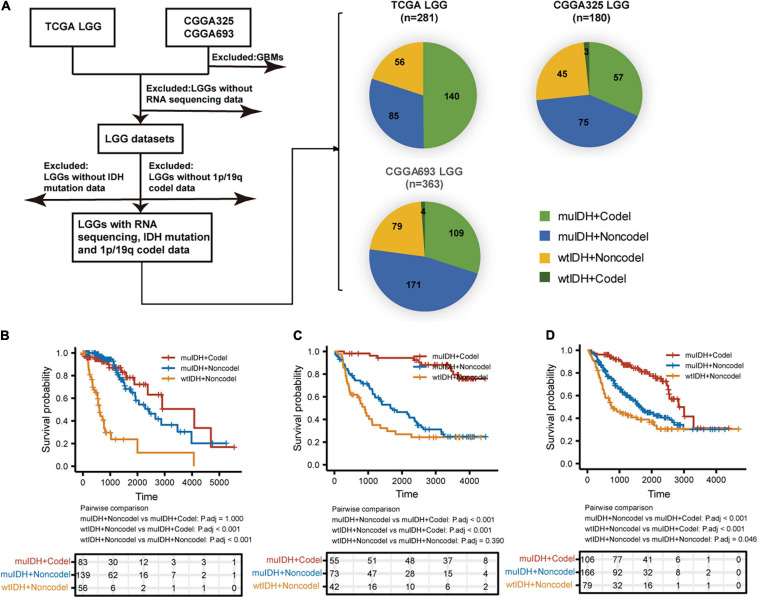
Sample and data collection in this study. **(A)** Only LGGs with RNA-sequencing data, IDH mutation, and 1p19q codel status information in TCGA and CGGA datasets were included in this study. **(B–D)** Kaplan–Meier analysis of OS (overall survival) of patients with LGGs according to IDH mutation and 1p/19q codel status in TCGA LGG **(B)**, CGGA325 LGG **(C)**, and CGGA693 LGG **(D)** dataset.

A total of 824 LGGs with full set of RNA-sequencing data, IDH mutation and 1p/19q codel status from both TCGA (The Cancer Genome Atlas) LGG and CGGA (Chinese Glioma Genome Atlas), including CGGA_325 and CGGA_693, were analyzed in this study (Figure 1). In each LGG dataset, as LGGs with both IDH wildtype and 1p/19q codeletion (wtIDH + Codel) were very few (<1.7%), there were mainly three subgroups of LGGs: (1) IDH mutation and 1p/19q codeletion (muIDH + Codel); (2) IDH mutation and 1p/19q non-codeletion (muIDH + Non-codel); and (3) IDH wildtype and 1p/19q non-codeletion (wtIDH + Non-codel) (Figure 1A).

Next, we examined the survival of LGG patients of different subgroups. The patients with “wtIDH + Non-codel” had the worst prognosis among the three subgroups, while those with “muIDH + Codel” had the best prognosis (Figures 1B–D). Moreover, patients with “muIDH + Non-codel” have a better prognosis than patients with “wtIDH + Non-codel” (Figures 1B–D), and patients with “muIDH + Codel” have a better prognosis than patients with “muIDH + Non-codel” (Figures 1B–D). These results suggest that both IDH mutation and 1p/19q codeletion are markers of better prognosis in LGG patients.

### Correlation of Immune Phenotype With 1p/19q Codel in LGGs

**FIGURE 2:**
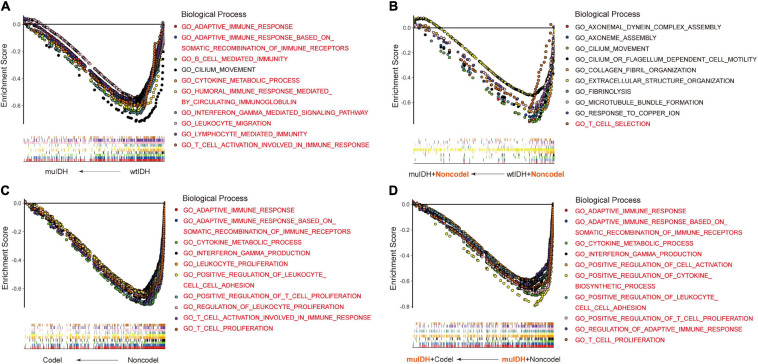
Gene Set Enrichment Analysis (GSEA) using the human biological processes (BPs) gene set. **(A)** The top ten BPs negatively associated with IDH mutation status in LGGs. **(B)** The top ten BPs negatively associated with IDH mutation status in LGGs with 1p/19q non-codel. **(C)** The top ten BPs negatively associated with 1p/19q codel status in LGGs. **(D)** The top ten BPs negatively associated with 1p/19q codel status in LGGs with IDH mutation. The immune-related BPs were marked in red.

Previous studies have demonstrated that IDH mutation and 1p/19q codel were early events during LGG tumorigenesis, however, patients with IDH mutation and 1p/19q codel had a significant better prognosis than their counterparts. To explore the underlying mechanism, we initially utilized RNA-sequencing data of LGG patients from the three above LGG datasets to identify the differential biological processes (BPs) according to IDH mutation and/or 1p/19q codel status using Gene Set Enrichment Analysis (GSEA), and only the common differential BPs in all three datasets were used for further analysis. It revealed that among the top ten negatively associated BPs for IDH mutation, nine were immune-related BPs (Figure 2A and Supplementary Table 1D), such as “B cell mediated immunity” (NES = −2.04, FDR q-val = 0.0020 in TCGA LGG), “interferon gamma mediated signaling pathway” (NES = −2.03, FDR q-val = 0.0015 in TCGA LGG), “lymphocyte mediated immunity”

(NES = −1.91, FDR q-val = 0.0053 in TCGA LGG), and “adaptive immune response” (NES = −1.90, FDR q-val = 0.0060 in TCGA LGG) (Figure 2A and Supplementary Table 1A,D), which was consistent with previous findings (Amankulor et al., 2017; Qian et al., 2018). To exclude the influence of 1p/19q codeletion status on the analysis, differential BPs were further analyzed between “muIDH + Non-codel” LGGs and “wtIDH + Non-codel” LGGs. It showed that only one of the top ten negatively associated BPs for IDH mutation in 1p/19q non-codel LGGs were immune-related BPs, which was “T cell selection” (NES = −1.78, FDR q-val = 0.0702 in TCGA LGG) (Figure 2B and Supplementary Tables 2A,D). These results suggested that rather than IDH mutation, 1p/19q codel contributed more to immune regulation in LGGs. Indeed, GSEA revealed that all top ten negatively correlated BPs for 1p/19q codel in LGGs were immune-related (Figure 2C and Supplementary Table 3D), such as “cytokine metabolic process” (NES = −2.42, FDR q-val = 0 in TCGA LGG), “adaptive immune response” (NES = −2.37, FDR q-val = 0 in TCGA LGG), “T cell proliferation” (NES = −2.36, FDR q-val = 0 in TCGA LGG), “T cell activation involved in immune response” (NES = −2.34, FDR q-val = 0 in TCGA LGG), and “interferon gamma production” (NES = −2.32, FDR q-val = 0 in TCGA LGG) (Figure 2C and Supplementary Table 3A). To exclude the influence of IDH mutation on the analysis, differential BPs was analyzed between “muIDH + Codel” LGG and “muIDH + Non-codel” LGGs. It showed that all top ten negatively correlated BPs for 1p/19q codel status in IDH mutation LGGs were immune-related BPs (Figure 2D and Supplementary Table 4D), such as positive regulation of cytokine biosynthetic process (NES = −2.46, FDR q-val = 0 in TCGA LGG), cytokine metabolic process (NES = −2.42, FDR q-val = 0), T cell proliferation (NES = −2.34, FDR q-val = 0), positive regulation of T cell proliferation (NES = −2.33, FDR q-val = 0 in TCGA LGG), adaptive immune response (NES = −2.32, FDR q-val = 0 in TCGA LGG), interferon gamma production (NES = −2.29, FDR q-val = 0 in TCGA LGG) (Figure 2D and Supplementary Table 4A). Taken together, these results indicate that 1p/19q codel is associated with immune-related phenotypes in LGGs.

### Low Level of Tumor-Infiltrating Immune Cells in 1p/19q Codel LGG

**FIGURE 3:**
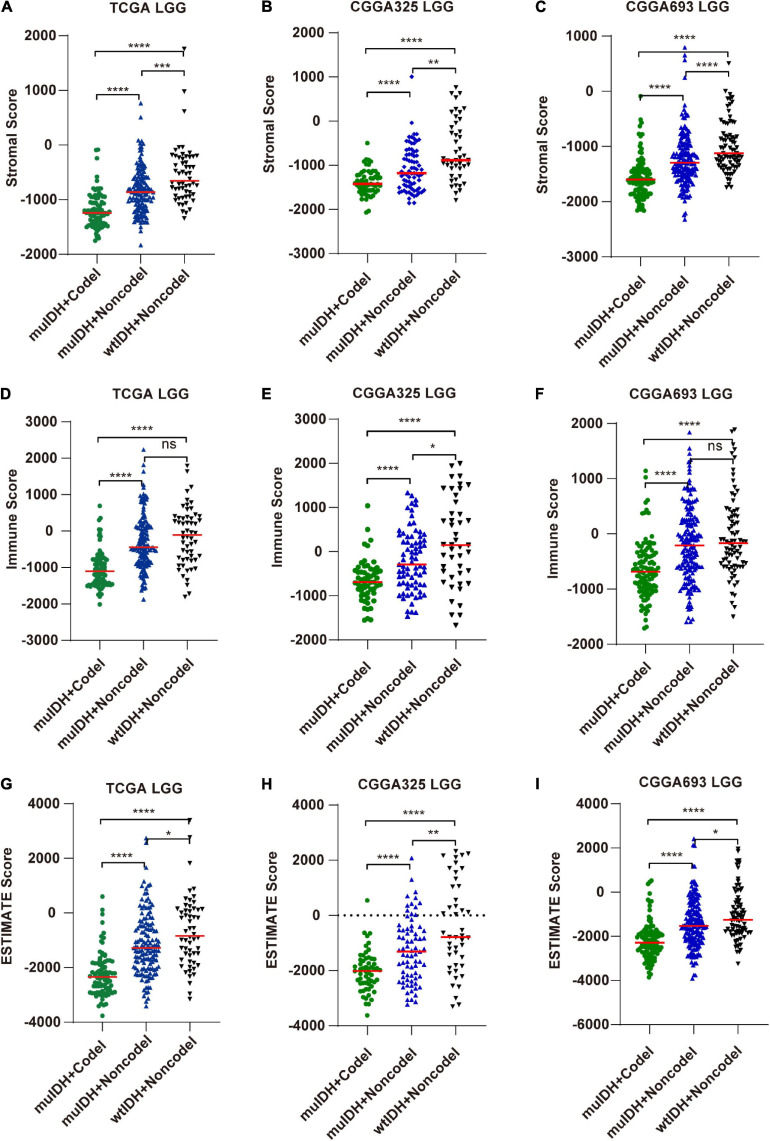
Estimating the infiltrating level of stromal cells, immune cell, and tumor purity in LGG using ESTIMATE algorithm. **(A–C)** Stromal score (positively correlated with the level of stromal cell in tumor) of samples in different subgroups in TCGA LGG (A), CGGA325 LGG **(B)**, and CGGA693 LGG **(C)**. **(D–F)** Immune score (positively correlated with immune cell infiltration in tumor) of samples in different subgroups in TCGA LGG (D), CGGA325 LGG **(E)**, and CGGA693 LGG **(F)**. **(G–I)** ESTIMATE score (negatively correlated with tumor purity) of samples in different subgroups of LGGs in TCGA LGG **(G)**, CGGA325 LGG **(H)**, and CGGA693 LGG **(I)**. Each symbol represents an individual patient. The number of patients in each subgroup was shown in Figure 1. The mean scores are indicated by red lines. *P* values are inferred from a two-sided Student’s *t*-test or Mann-Whitney test. *p < 0.05, **p < 0.01, ***p < 0.001, ****p < 0.0001.

The tumor microenvironment (TME), consisting of stromal cells, immune cells, and other factors, is closely related to immune evasion in tumors (Labani-Motlagh et al., 2020). By closely interacting with tumor cells, these cells participate in all stages of tumor progression and are associated with the response to immunotherapy, consequently affecting patients’ survival (Guo and Deng, 2018). Immune cell migration and immune infiltration are directed by cytokines (Kohli et al., 2021). However, biological processes related to cytokine were impaired in 1p/19q codel LGGs (Figures 2C,D). Therefore, we speculate that 1p/19q codel may affect immune cell infiltration. We used the “ESTIMATE” algorithm to infer tumor purity (negatively correlated with ESTIMATE score), infiltration level of stromal cells (positively correlated with stromal score), and immune cells (positively correlated with immune score) in LGGs using gene expression data in the above three LGG datasets. The average stromal/immune/ESTIMATE score were significantly lower in both IDH mutation (muIDH) LGGs and 1p/19q codel (Codel) LGGs compared to IDH wildtype (wtIDH) LGGs (Supplementary Figures 1A–C,G–I, and M-O) and 1p/19q non-codel (Non-codel) LGGs (Supplementary Figures 1D–F,J–L and P-R), respectively. And an analysis focusing on the role of 1p/19q codel showed that the average stromal/immune/ESTIMATE scores were significantly lower in muIDH + Codel LGGs than muIDH + Non-codel LGGs in all three datasets (Figure 3). Moreover, stromal/immune/ESTIMATE scores were lower in wtIDH + Codel LGGs than wtIDH + Non-codel LGGs in all datasets, despite the lack of statistically significant differences due to the small sample numbers of wtIDH + Codel LGGs (data not shown). However, we did not observe significant differences in the average immune score between muIDH + Non-codel LGGs and wtIDH + Non-codel LGGs in TCGA LGG and CGGA693 LGG datasets (Figure 3), indicating that IDH mutation might contribute little to immune infiltration. These analyses showed that 1p/19q codel was strongly associated with lower infiltration level of stromal/immune cells and higher tumor purity in LGGs.

Infiltrating level of tumor-infiltrating immune cells (TIICs) could profoundly influence tumor immune evasion and tumor progression, as well as the success of anti-cancer therapies. We then quantified the TIICs in all three LGG datasets through two other widely-used bioinformatics tools: “Timer” (Tumor Immune Estimation Resource) (Li et al., 2017) and “xCell” (Aran et al., 2017).

**FIGURE 4:**
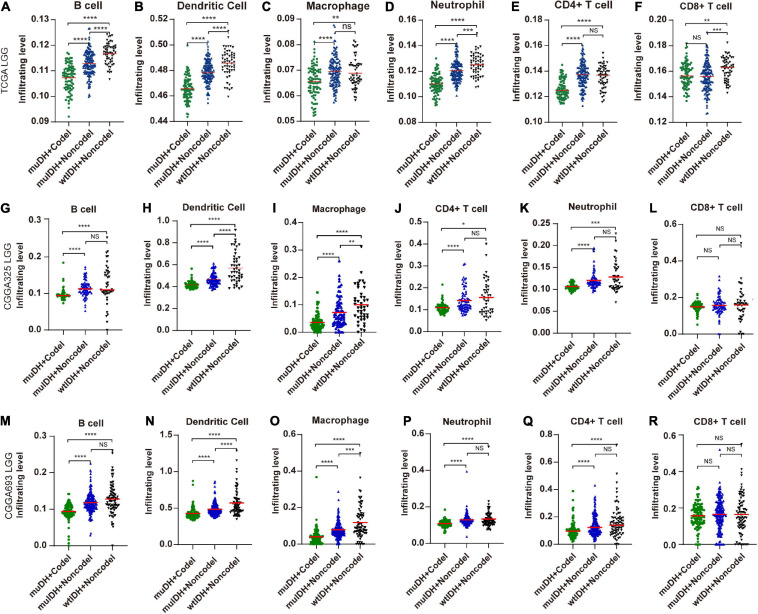
Estimating the infiltrating level of six kinds of immune cells in LGG using TIMER tool. **(A–F)** Infiltrating level of six kinds of immune cell in different subgroups in TCGA LGG. **(G–L)** Infiltrating level of six kinds of immune cell in different subgroups in CGGA325 LGG. **(M–R)** Infiltrating level of six kinds of immune cell in different subgroups in CGGA693 LGG. Each symbol represents an individual patient. The number of patients in each subgroup was shown in Figure 1. The mean scores are indicated by red lines. *P* values are inferred from a two-sided Student’s *t*-test or Mann-Whitney test. *p < 0.05, **p < 0.01, ***p < 0.001, ****p < 0.0001.

“TIMER” could estimate the abundances of six types of immune cells, including B-cells, CD4^+^ T-cells, CD8^+^ T-cells, dendritic cells, macrophages, and neutrophils, based on a list of immune-specific markers (Li et al., 2017). It showed here that infiltration level of B-cells, CD4^+^ T-cells, dendritic cells, macrophages, and neutrophils in muIDH + Codel LGGs were significantly lower than those in muIDH + Non-codel LGGs, but not for CD8^+^ T-cells (Figure 4). In addition, the infiltrating levels of B-cells, CD4^+^ T-cells, dendritic cells, macrophages, and neutrophils in wtIDH + Codel LGGs were also lower than in wtIDH + Non-codel LGGs, despite the lack of statistically significant differences due to the small sample numbers of wtIDH + Codel LGGs (Figure 4). However, only infiltrating level of dendritic cells was significantly lower in muIDH + Non-codel LGGs than wtIDH + Non-codel LGGs in all three datasets.

**FIGURE 5:**
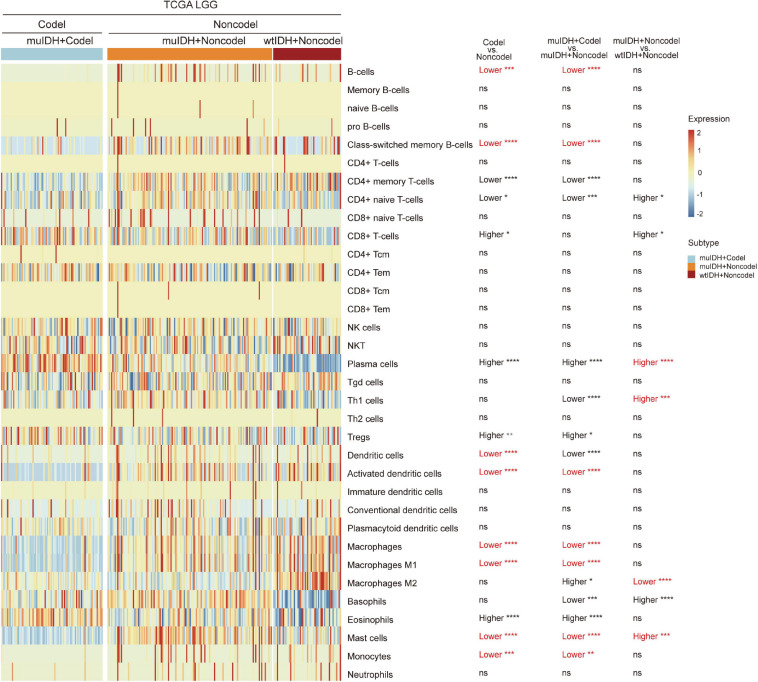
Estimating the infiltrating level of 34 kinds of immune cells in TCGA LGG using xCell tool. The heatmap shows the infiltrating levels of immune cells in different subgroup of LGGs in TCGA LGG. The significant differences confirmed in both CGGA325 and CGGA693 LGG datasets were marked in red. The number of patients in each subgroup was shown in Figure 1. *P* values are inferred from a two-sided Student’s *t*-test or Mann-Whitney test. *p < 0.05, **p < 0.01, ***p < 0.001, ****p < 0.0001.

“xCell” could estimate the abundance scores of 64 cell types, including 34 kinds of immune cells and other cells, based on a novel compendium of 489 gene sets extracted from large-scale expression data from different projects and studies (Aran et al., 2017). xCell analysis based on 1p/19q status showed that the infiltrating level of 7 kinds of immune cells was significantly lower in muIDH + Codel LGGs than in muIDH + Non-codel LGGs in all three LGG datasets, including B-cells, class-switched memory B-cells, activated dendritic cells, macrophages, macrophages M1, mast cells and monocytes (Supplementary Figures 2, 3). However, analysis based on IDH mutation status showed that only the infiltrating level of “Macrophage M2” was significantly lower in muIDH + Non-codel LGGs than in wtIDH + Non-codel LGGs in all three LGG datasets (Figure 5 and Supplementary Figures 2, 3), and the levels of plasma cells, Th1 cells, and mast cells were even significantly higher in muIDH + Non-codel LGGs than in wtIDH + Non-codel LGGs in all three LGG datasets (Figure 5 and Supplementary Figures 2, 3).

**FIGURE 6:**
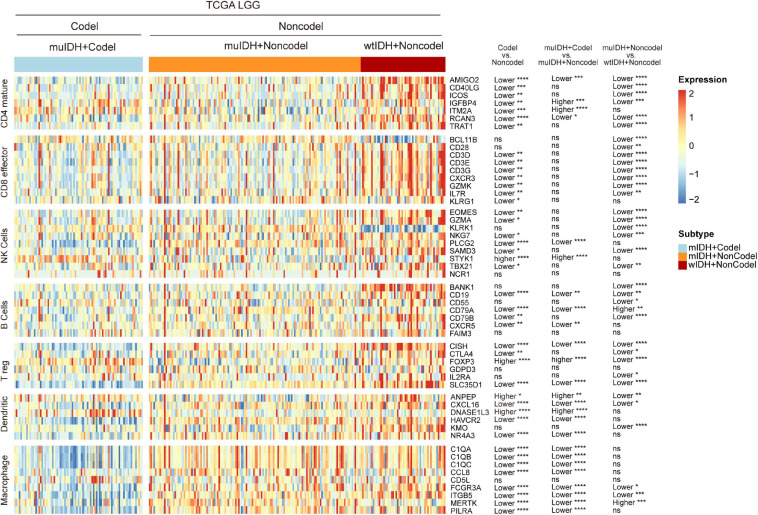
Expression of marker genes for immune cells in TCGA LGG. The heatmap shows the expression levels of marker genes for CD4^+^ T cells, effector CD8^+^ T cells, NK cells, B cells, T-reg cells, dendritic cells, and macrophages among different subgroup LGGs in TCGA LGG. *P* values are inferred from a two-sided Student’s *t*-test or Mann-Whitney test. The number of patients in each subgroup was shown in Figure 1. *p < 0.05, **p < 0.01, ***p < 0.001, ****p < 0.0001.

To confirm the changes in immune cell infiltration analyzed through ESTIMATE, TIMER, and xCell, we analyzed the expression of marker genes for immune cells. These genes are specifically expressed in each type of immune cell and not in other immune cells or epithelial cells (Davoli et al., 2017). The majority of these genes showed lower expression in 1p/19q codel LGGs than non-codel cohorts (Figure 6 and Supplementary Figures 4, 5). And the expression of marker genes specific for B cells and macrophages was significantly reduced in muIDH + Codel LGGs relative to muIDH + Non-codel LGGs (Figure 6 and Supplementary Figures 4, 5). Thus, we infer from these cell type specific gene expression changes that the infiltrating levels of these types of immune cells expressing the marker genes are correspondingly reduced.

Taken together, these results show that 1p/19q codel and IDH mutations contribute differently to the immune cell infiltration in LGGs, and 1p/19q codel is associated with low infiltrating level of immune cells in LGGs.

### Reduced Expression of Immune Checkpoint Genes in 1p/19q Codel LGGs

**FIGURE 7:**
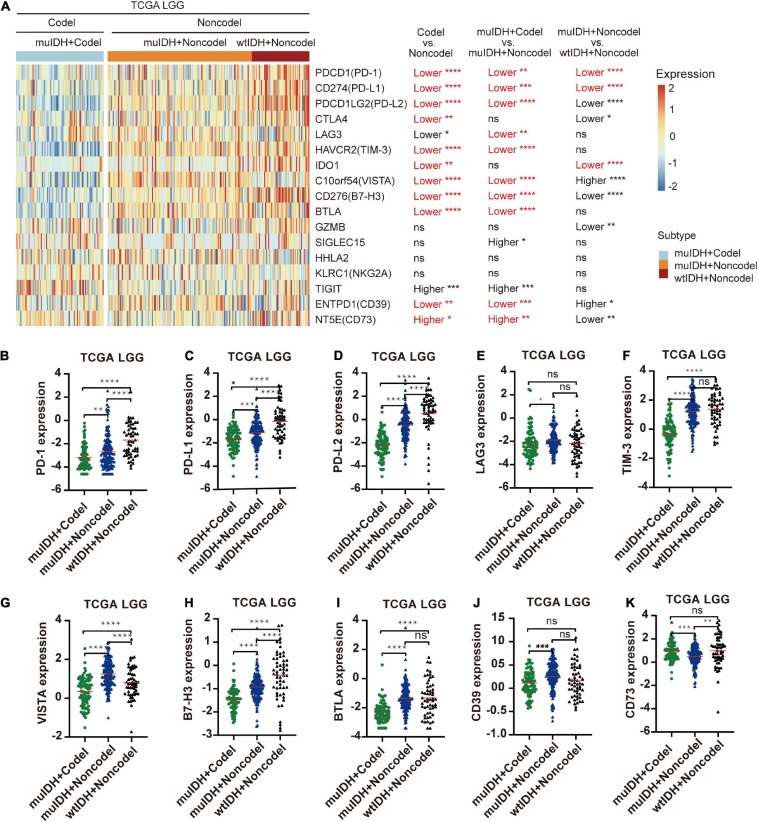
Differentially expressed immune checkpoint genes among different subgroups. **(A)** The heatmap shows the expression levels of immune-related genes on chromosome 1p/19q among different subgroup LGG patients in TCGA LGG. The significant differences confirmed in both CGGA325 and CGGA693 LGG were marked in red. **(B–K)** The relative mRNA expression of PD-1 **(B)**, PD-L1 **(C)**, PD-L2 **(D)**, LAG3 **(E)**, TIM-3 **(F)**, VISTA **(G)**, B7-H3 **(H)**, BTLA **(I)**, CD39 **(J)**, and CD73 **(K)** in TCGA LGG. The mean mRNA expression was indicated by red lines. *P* values are inferred from a two-sided Student’s *t*-test or Mann-Whitney test. The number of patients in each subgroup was shown in Figure 1. *p < 0.05, **p < 0.01, ***p < 0.001, ****p < 0.0001.

The immune checkpoint plays a crucial role in tumor evasion (Kok, 2020). Overexpression of immune checkpoint genes, such as PD-L1/L2, can cause T cell exhaustion and evade immune surveillance, thus promote tumor progression and is associated with worse survival. We then analyzed the association between 1p/19q codel and the expression of multiple immune checkpoint genes. Expression of ten immune checkpoint genes, including PD-1, PD-L1, PD-L2, CTLA4, TIM-3, IDO1, VISTA, B7-H3, BTLA, and CD39, were significantly lower in 1p/19q codel LGG compared to 1p/19q non-codel LGG in all three LGG datasets (Figure 7A and Supplementary Figure 6), only the expression of CD73 was significantly higher in 1p/19q codel LGGs compared to 1p/19q non-codel LGGs. What is more, the expression of nine immune checkpoint genes, including PD-1, PD-L1, PD-L2, LAG3, TIM-3, VISTA, B7-H3, BTLA, and CD39, were also significantly lower in muIDH + Codel LGG than muIDH + Non-codel LGGs in all three LGG datasets (Figures 7A–K and Supplementary Figure 6). In addition, expression of three immune checkpoint genes, including PD-1, PD-L1, and IDO1, were significantly lower in muIDH + Non-codel LGGs than wtIDH + Non-codel LGGs in all three LGG datasets (Figure 7A and Supplementary Figure 6). These results suggested that 1p/19q codel LGGs with lower expression of immune checkpoint genes might be more susceptible to immune surveillance compared with 1p/19q non-codel LGGs, and so did the IDH mutation. It was particularly noteworthy that the expression of PD1 and PD-L1 in muIDH + Codel subgroup was the lowest among the three subgroups of LGGs (Figures 7B,C and Supplementary Figure 6), which may account for the best prognosis (Weller et al., 2012). Taken together, these results show that 1p/19q codel and IDH mutation contribute differently to the expression of immune checkpoint genes in LGGs, and 1p/19q codel is associated with low expression of multiple immune checkpoint genes in LGGs.

### Immune-Related Genes on Chromosome Arms 1p and 19q

**FIGURE 8:**
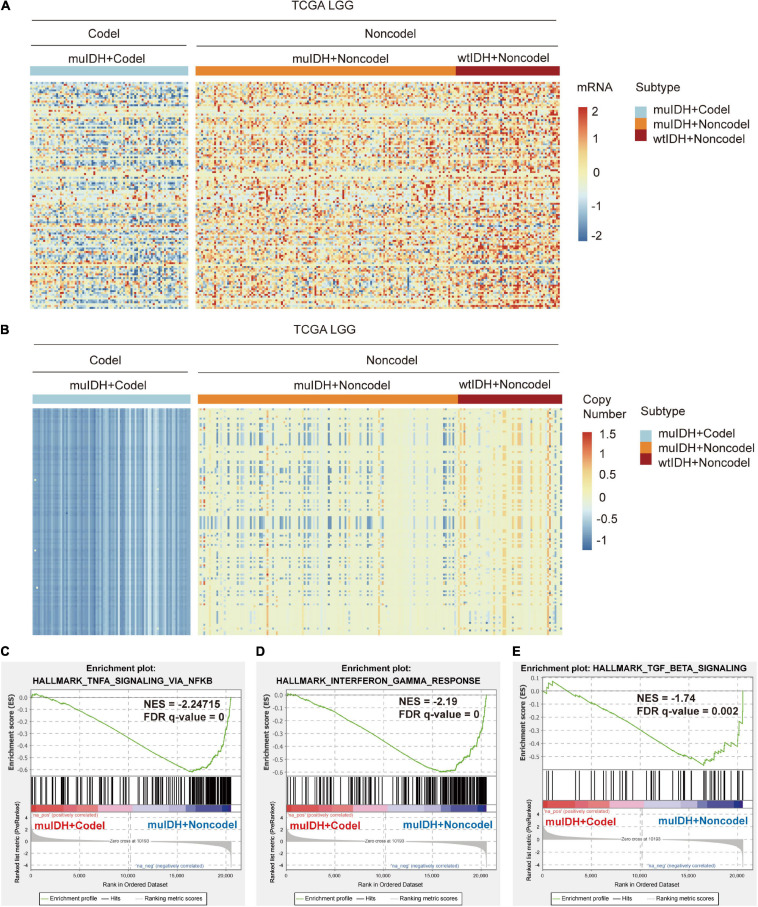
Expression and copy number variation of immune-related genes on chromosome 1p/19q. **(A)** Heatmap of differentially expressed immune-related genes in LGG patients among different subgroups in TCGA LGG. **(B)** Heatmap of copy number variation of immune-related genes in LGG patients among different subgroups in TCGA LGG. **(C–E)** Gene set enrichment analysis indicates a significantly reduced “TNFA SIGNALING VIA NFKB” **(C)**, “INTERFERON GAMMA RESPONSE” **(D)**, and “TGF BETA SIGNALING” **(E)** in the cases of LGGs with 1p/19q codel.

Based on the immune-related gene list in Immport database (Bhattacharya et al., 2018), there are 127 immune genes located on chromosome 1p/19q, including 65 genes on chromosome 1p and 62 genes on chromosome 19q (Supplementary Table 5). These genes are distributed in 13 immune categories, including “Antigen Processing and Presentation,” “Antimicrobials,” “BCR signaling pathway,” “Chemokine receptors,” “Chemokines,” “Cytokine receptors,” “Cytokines,” “Interleukins,” “Interleukins receptor,” “Natural killer cell cytotoxicity,” TCR signaling Pathway,” “TGF-β family member,” and “TNF family members receptors” (Supplementary Table 5). The immune-related genes’ copy number and mRNA expression were significantly lower in 1p/19q codel LGGs than in non-codel LGGs (Figures 8A,B and Supplementary Figure 7).

**FIGURE 9:**
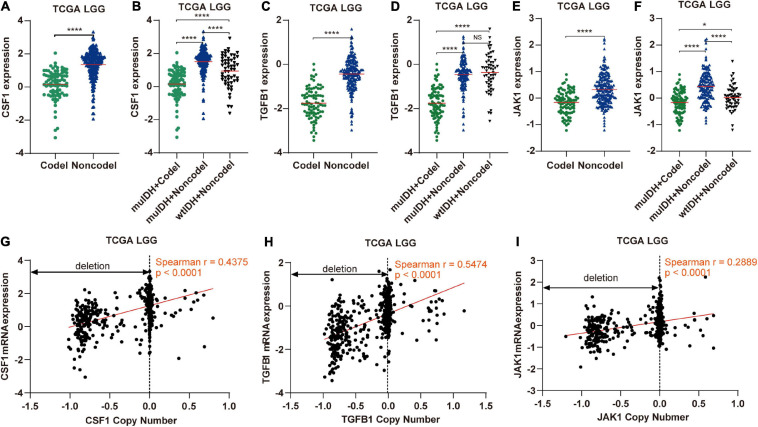
The expression and copy number of CSF1, TGFB1, and JAK1 among different subgroups classified by IDH mutation and 1p/19q codel status in TCGA LGG. **(A,C,E)** The expression of CSF1, TGFB1, and JAK1 in 1p/19q codel and 1p/19q non-codel LGGs in TCGA LGG dataset. **(B,D,F)** The expression of CSF1, TGFB1, and JAK1 in different subgroups. The mean mRNA expression was indicated by red lines. *P* values are inferred from a two-sided Student’s *t*-test or Mann-Whitney test. (*p < 0.05, ****p < 0.0001). **(G–I)** Correlation analysis between copy number alteration and mRNA expression of CSF1, TGFB1, and JAK1 in TCGA LGG, respectively.

Reduced expression of these genes would lead to dysregulated immune behaviors and defective immune-related signaling pathways. Firstly, immune cell infiltration. Previous reports have shown that chemokine and cytokine could stimulate immune cell migration to wounded sites and drive immune cell infiltration in tumor. In this regard, 10 chemokine/chemokine receptors related genes and 75 cytokines/cytokine receptors-related genes located on chromosome 1p/19q (Supplementary Table 5) were low-expressed in 1p/19q codel LGGs, which was in agreement with the low level of immune cell infiltration in 1p/19q codel LGGs. Secondly, the expression of immune checkpoint genes. For example, the TGF-β signaling pathway plays a central role in immune regulation in various tumors, including gliomas. It could upregulate the expression of some immune checkpoint genes, including TIM-3, B7-H3, PD-1, and PD-L1/L2 (Wiener et al., 2007; Song et al., 2014; Zhou et al., 2016; Tauriello et al., 2018). Two members of TGF-β Family, including TGFB1 and TGFBR3, are located on chromosome 1p/19q and are low-expressed in 1p/19q codel LGGs (Table 1 and Supplementary Table 5 and Figures 9C,D). GSEA using the HALLMARK gene sets showed that gene sets related to “TGF BETA SIGNALING” were depleted in 1p/19q codel LGG (Figure 8E). Besides, TNF-α and INF-γ signaling could also induce the expression of immune checkpoint genes, such as CD80/CD86, MHC-II, GAL-9, and PD-L1/L2 expression (Steimle et al., 1994; Li et al., 1996; Yokozeki et al., 1997; Imaizumi et al., 2002; Giroux et al., 2003). GSEA using the HALLMARK gene sets showed that gene sets related to “TNFA SIGNALING VIA NFKB” and “INTERFERON GAMMA RESPONSE” were also depleted in 1p/19q codel LGG (Figures 8C,D). Seven members of the TNF Family and JAK1, an essential gene in interferon-γ signaling, are located on chromosome 1p/19q and low expressed in 1p/19q codel LGG (Supplementary Table 5 and Figures 9E,F).

**TABLE 1:** Positive correlation between copy number and mRNA expression of immune-related genes mapped on chromosome 1p and 19q.

**Gene Symbol**	**Cytogenetic band**	**Spearman R**	***P* Value**	**Gene Symbol**	**Cytogenetic band**	**Spearman R**	***P* Value**
AKT2	19q13.2	0.6532	< 0.0001	JAK1	1p31.3	0.2988	< 0.0001
ANGPTL3	1p31.3	0.203	0.0006	JUN	1p32.1	0.4343	< 0.0001
ANGPTL7	1p36.22	0.1505	0.0115	KIR2DL4	19q13.42	0.1175	0.0491
ARTN	1p34.1	0.3591	< 0.0001	LCK	1p35.2	0.2068	0.0005
BCL10	1p22.3	0.611	< 0.0001	LHB	19q13.33	0.1889	0.0015
BCL3	19q13.32	0.4625	< 0.0001	LILRB3	19q13.42	0.2968	< 0.0001
BMP8A	1p34.3	0.1843	0.0019	LTBP4	19q13.2	0.2149	0.0003
BMP8B	1p34.2	0.4782	< 0.0001	MIA	19q13.2	0.4909	< 0.0001
C5AR1	19q13.32	0.3822	< 0.0001	MPL	1p34.2	0.3531	< 0.0001
CBLC	19q13.32	0.1291	0.0305	NFKBIB	19q13.2	0.6871	< 0.0001
CD79A	19q13.2	0.2645	<0.0001	NFYC	1p34.2	0.6512	<0.0001
CDC42	1p36.12	0.6931	< 0.0001	NPPB	1p36.22	0.1901	0.0014
CGB5	19q13.33	0.176	0.0031	NR1H2	19q13.3	0.7085	< 0.0001
CGB7	19q13.33	0.2682	< 0.0001	NRAS	1p13.2	0.5729	< 0.0001
CLEC11A	19q13.33	0.4331	< 0.0001	PAK4	19q13.2	0.6664	< 0.0001
CORT	1p36.22	0.1253	0.0358	PIK3CD	1p36.22	0.4548	< 0.0001
CSF1	1p13.3	0.4255	<0.0001	PIK3R3	1p34.1	0.2809	<0.0001
CSF3R	1p34.3	0.5089	< 0.0001	PLA2G2A	1p36.13	0.2197	0.0002
CXCL17	19q13.2	0.1186	0.0469	PLAUR	19q13	0.4892	< 0.0001
CYR61	1p22.3	0.3810	< 0.0001	PRDX1	1p34.1	0.5090	< 0.0001
EDN2	1p34.2	0.1284	0.0314	PSMC4	19q13.11	0.7142	< 0.0001
FAM19A3	1p13.2	0.2913	<0.0001	PSMD8	19q13.2	0.6794	<0.0001
FCGRT	19q13.33	0.5674	< 0.0001	PTAFR	1p35.3	0.4787	< 0.0001
FLT3LG	19q13.33	0.5512	< 0.0001	RBP7	1p36.22	0.1241	0.0376
FPR1	19q13.41	0.4101	< 0.0001	RELB	19q13.32	0.5249	< 0.0001
FPR2	19q13.41	0.2019	0.0007	S1PR1	1p21.2	0.3356	< 0.0001
GBP2	1p22.2	0.4209	<0.0001	SDC3	1p35.2	0.1554	0.0091
GMFG	19q13.2	0.4936	< 0.0001	SORT1	1p13.3	0.3040	< 0.0001
GPI	19q13.11	0.3549	< 0.0001	TGFB1	19q13.2	0.5559	< 0.0001
GPR77	19q13.32	0.2989	< 0.0001	TGFBR3	1p22.1	0.2579	< 0.0001
HAMP	19q13.12	0.4747	< 0.0001	TNFRSF14	1p36.32	0.2589	< 0.0001
HCST	19q13.12	0.4955	< 0.0001	TNFRSF1B	1p36.22	0.4593	< 0.0001
HDAC1	1p35.2	0.6361	< 0.0001	TNFRSF25	1p36.31	0.2626	< 0.0001
IL11	19q13.42	0.2047	0.0006	TNFRSF8	1p36.22	0.4587	< 0.0001
IL28RA	1p36.11	0.5157	<0.0001	TXLNA	1p35.2	0.6863	<0.0001
INSL5	1p31.3	0.3957	< 0.0001	TYROBP	19q13.12	0.5035	< 0.0001
IRF3	19q13.33	0.7338	< 0.0001	VAV3	1p13.3	0.2123	0.0003
ISG15	1p36.33	0.258	< 0.0001	VCAM1	1p21.2	0.5547	< 0.0001

Next, by analyzing the correlation between DNA copy number and mRNA expression of these immune-related genes on chromosome 1p/19q, we found that the mRNA expression was correlated positively with the copy number in 76 immune-related genes (Table 1), which suggests that copy number loss caused by 1p/19q codeletion is one of the main reasons for the reduced mRNA expression of these genes. We took three 1p/19q genes, CSF1, TGFB1, and JAK1, as examples to analyze their expression and relationship with copy number variation (CNV). As shown in Figures 9A–F and S8, the mRNA expression of CSF1, TGFB1, and JAK1 were significantly lower in 1p/19q codel LGGs than those in 1p/19q non-codel LGGs, regardless of the status of IDH. In addition, their expression in wtIDH + Codel LGGs was lower than that in wtIDH + Non-codel LGGs, although the difference was not significant due to the small number of wtIDH + Codel samples (data not shown). Moreover, the copy number of CSF1, TGFB1, and JAK1 positively correlated with their expression (Figures 9G–I).

Taken together, these results show that 1p/19q codeletion reduces the copy number of immune-related genes on chromosome 1p and 19q, then decreases the expression of these genes. And these genes are involved in critical signaling pathways regulating immune response and immune cell infiltration, such as “TGF BETA SIGNALING,” “TNFA SIGNALING VIA NFKB,” and “INTERFERON GAMMA RESPONSE.”

## Discussion

A critical unanswered question in cancer research is how tumors evolve and how genetic/epigenetic alterations shape the evolution. IDH1/2 mutation and chromosome 1p/19q codel are the two most common genetic changes in LGGs, representing driver events during glioma tumorigenesis, and are associated with better survival rates in glioma. However, the underlying mechanisms responsible for these different clinical outcomes are still not completely clear. To demonstrate that differences in genomic alterations among different types of gliomas significantly contribute to these outcomes, discriminating the effects of IDH mutation and 1p/19q codel will be needed definitively.

The effect of 1p/19q codel on the development of gliomas is challenging to study for several reasons. First and foremost, it is almost impossible to delete or restore the chromosome 1p/19q in glioma cell lines like working on a specific gene. Second, mouse models of brain tumors are not applicable for 1p/19q codeletion research. Genetically engineered mice (GEM) are unsuitable because of different distribution of genes on chromosomes between humans and mice. Furthermore, the xenograft tumor models may not be able to simulate the effect of 1p/19q codel on the infiltration of immune cells, because the interaction between human glioma cells and mouse immune cells is likely to be different from that between human glioma cells and human immune cells.

By exerting anti-tumor to pro-tumor activities, TIICs can profoundly influence tumorigenesis. Therefore, quantification of TIICs will help to reveal the role of the immune system in tumor development and immune evasion. Previous reports had shown that IDH mutation gliomas had lower lymphocyte (CD3^+^; PD1^+^) infiltration (Berghoff et al., 2017) and/or CD8^+^ cytotoxic T cell infiltration (Kohanbash et al., 2017). And IDH mutation in mice caused down-regulation of leukocyte chemotaxis, resulting in repression of tumor-associated immune cells infiltration (Amankulor et al., 2017; Kohanbash et al., 2017; Bunse et al., 2018). However, the 1p/19q codel was found in 38-61% of IDH mutant glioma (Figure 1). Thus, whether 1p/19q codel also affected the infiltration of immune cells remained unclear. In this study, we quantified TIICs from RNA sequencing data of 824 LGG cases from three independent LGG datasets using bioinformatics approaches and analyzed their relationship with 1p/19q codel and IDH mutation status, respectively. Both IDH mutation and 1p/19q codel were associated with low infiltrating levels of TIICs in LGG. The latter contributed more, and they also showed different effects on immune cell infiltration. For example, the infiltrating level of seven kinds of immune cells, such as B cells and macrophages, was significantly lower in muIDH + Codel LGG than in muIDH + Non-codel LGGs. However, only the infiltrating level of M2 Macrophages was markedly lower in muIDH + Non-codel samples than in wtIDH + Non-codel samples (Figure 5).

Immune cell infiltration in tumors is often stimulated by chemokines and cytokines (Franciszkiewicz et al., 2012; Domingues et al., 2016). A total of 41 genes encoding cytokines and chemokines are located on chromosome 1p/19q, and their expression was significantly lower in 1p/19q codel tumor than in 1p/19q non-codel LGG due to copy number deletion. For example, TGF-β1, a secreted cytokine encoded by TGFB1 which is located on chromosome 19q, could enhance immune cell infiltration in tumor (Yang et al., 2010). CSF1 is another kind of cytokine, which is located on chromosome 1p13.3, could promote the infiltration and survival of tumor associated macrophages (TAMs) (Dwyer et al., 2017). And its low expression in 1p/19q codel LGGs maybe account for the low infiltrating level of macrophage in 1p/19q codel glioma. Of course, the decreased immune cell infiltration in 1p/19q codel LGG could not only be attributed to these two cytokines, but may also be caused by many other cytokines/chemokines, or even other proteins. For example, the expression of JAK1, which is located on chromosome 1p31.3, also had significant positive correlations with infiltrating levels of CD8^+^ T cells, CD4^+^ T cells, macrophages, neutrophils, and dendritic cells in cancer (Chen B. et al., 2019).

Historically, the immune infiltrates of tumor could be investigated with immunohistochemistry (IHC), immune fluorescence (IF), and flow cytometry. However, in such experiments, the number of cases that can be used to verify is small, and the types of immune cells that can be detected at the same time are also limited due to cross-reactivity between antibodies. Therefore, the quantitation of the immune infiltrates in a large series of patients was time-consuming and complex. With the development of next-generation sequencing (NGS) technologies, a large number of sequencing data have been generated, such as TCGA, which can be used to describe tumor microenvironment. The composition of TIICs can be characterized from RNA-sequencing data using computational approaches. In the present study, we analyzed the relationship among 1p/19q codel status, IDH mutation status, and RNA data in a total of 824 LGG cases from three independent datasets using three kinds of widely used bioinformatics tools: ESTIMATE, TIMER, and xCell. All the results from these tools pinpointed a significant decrease of immune infiltrating cells in 1p/19q codel LGGs. And the expression of marker genes specifically expressed in immune cells showed lower expression in 1p/19q codel LGGs than non-codel cohorts. In a recent study, Lin et al. (2021) estimated the infiltration level of immune cells in LGGs used ssGSEA (single sample gene set enrichment analysis). They found that the infiltration level of immune cells gradually increased in “IDH WT + 1p/19q non-codel”, “IDH mutation + 1p/19q non-codel”, and “IDH mutation + 1p/19q codel” LGGs. Some kinds of immune cells estimated by this method were the same as ours, such as NK T cells, NK cells, monocytes, eosinophils, neutrophils, Immature dendritic cells, plasmacytoid dendritic cells, activated dendritic cells, mast cells, and macrophages. We estimated the infiltration level of other 24 kinds of immune cells, such as B cells, CD4^+^ T-cells, CD8^+^ T-cells, plasma cells, Treg cells, dendritic cells, M1 macrophages, M2 macrophages and so on. In addition, we analyzed the relationship between IDH mutation, 1p/19q codel and the level of immune cell infiltration separately. We found that 1p/19q codeletion was more closely associated with the level of immune cell infiltration. Lin et al. (2021) found that the expression of immune checkpoint (PD-1, PD-L1, LAG3, and TIM-3) and HLA (human leukocyte antigen) genes gradually decreased in “IDH WT + 1p/19q non-codel”, “IDH mutation + 1p/19q non-codel”, and “IDH mutation + 1p/19q codel” LGGs. Our study showed that more kinds of immune checkpoint gene expression, such as PD-L2, VISTA, B7-H3, and BTLA, also decreased in IDH mutation and 1p/19 codeletion LGGs. These results indicated that patients with “IDH WT + 1p/19q non-codel” LGGs might benefit more from immune checkpoint blockade therapy. Lin et al. (2021) analyzed the differences of KEGG pathways and GO-BPs only between “IDH WT + 1p/19q non-codel” and “IDH mutation + 1p/19q codel” LGGs. In our study, we analyzed the differences of GO-BPs between “IDH WT + 1p/19q non-codel” and “IDH mutation + 1p/19q non-codel” LGGs, and between “IDH mutation (+1p/19q non-codel” and “IDH mutation (+1p/19q codel” LGGs. The result indicated that 1p/19q codeletion status was more closely associated with immune-related BPs in LGGs. Moreover, our analysis also revealed that 1p/19q codel was negatively correlated with three critical hallmarks related to immune infiltration, including TGF-(β signaling, TNF-(α signaling, and INF-(γ signaling. We further investigated the molecular mechanism underlying this association. Our analysis showed that 1p/19q codeletion reduced the copy number of many immune-related genes on chromosome 1p and 19q, such as TGFB1, CSF1, and JAK1, which led to the reduction of their expression level. Aberrant expression of these genes has been reported to influence immune cell infiltration and tumor immune evasion (Wiener et al., 2007; Song et al., 2014; Zaretsky et al., 2016; Zhou et al., 2016; Tauriello et al., 2018). Thus, the results of the present study provide a possible candidate mechanism for this phenomenon. However, both our study and the Lin et al. (2021) study are based on bioinformatics analysis and require further experimental validation *in vitro* and/or *in vivo*.

It is noteworthy that the infiltrating level of immune cells on tumor progression is complex. They could exert anti-tumor to pro-tumor activities dependent on the types of immune cells that infiltrate and the tumor type. For example, tumor-infiltrating B cells could produce cytokines that activate IκB kinase α (IKKα) to promote the progress and recurrence of prostate cancer (Ammirante et al., 2013). Also, tumor-infiltrating B cells promoted human hepatocellular carcinoma (HCC) growth and invasiveness by directly interacting with liver cancer cells through the CD40/CD154 signaling pathway (Shao et al., 2014). However, high infiltration of B cells was associated with favorable outcomes in breast, cervical, and colorectal (Nedergaard et al., 2008; Schmidt et al., 2008; Berntsson et al., 2016). Some studies showed that B cells could induce and maintain beneficial antitumor activity, while others have found that B cells may exert pro-tumor functions (Wang et al., 2019). It has also been reported that higher dendritic cell infiltration correlated with either better or worse prognosis in different tumors. For example, patients with high levels of dendritic cells in colorectal cancer exhibited shorter disease-free and overall survival than those with low levels (Jochems and Schlom, 2011). Conversely, the presence of tumor-infiltrating dendritic cell was associated with regression of melanoma (Tran Janco et al., 2015). Thus, further research is required to investigate the effect of low infiltrating level of immune cells, such as B cells and dendritic cells, in 1p/19q codel LGGs on the progress of LGG and survival of LGG patients.

We showed here that the expression of multiple immune checkpoint genes was significantly lower in 1p/19q codel LGGs compared to 1p/19q non-codel LGGs. This may be mainly due to two reasons: (1) Low level of immune cells in 1p/19q codel LGG, which was discussed above. (2) Differentially expressed genes affecting immune regulation. For example, the “interferon γ signaling pathway”, which was impaired in 1p/19q codel LGGs, could upregulate the expression of PD-L1 and PD-L2 through interferon-γ-JAK1/JAK2-IRF1 axis (Garcia-Diaz et al., 2017). Loss of JAK1 led to instability of PD-L1 (Chan et al., 2019) and significantly reduced the number of NK cells (Witalisz-Siepracka et al., 2018). TGF-β could upregulate the expression of PD-1 and PD-L1 (Wiener et al., 2007; Song et al., 2014; Zhou et al., 2016). Loss of four TGF-β family members, especially for TGFB1, in 1p/19q codel LGGs would lead to decreased expression of these immune checkpoint genes, which was consistent with our results. Immune evasion is a necessary step in tumor evolution (Hanahan and Weinberg, 2011), and high expression of immune checkpoint genes often leads to immune evasion in tumor (Juneja et al., 2017). Additionally, high level of TIICs also induces immune evasion in tumor (Qian and Pollard, 2010), which indicated that 1p/19q non-codel LGGs were more likely to escape immune surveillance than 1p/19q codel LGGs. Actually, TGF-β, impaired in 1p/19q codel LGGs, could drive immune evasion in multiple tumors (Zhou et al., 2016; Tauriello et al., 2018). This might partly explain the better prognosis of LGG patients with 1p/19q codel. On the other hand, tumors with high expression of immune checkpoint genes are more likely to respond to corresponding immune checkpoint blockade (ICB) therapy. Their expression is currently used as a biomarker for ICB therapy (Topalian et al., 2012). For example, the detection of PD-L1 protein expression could reflect the response to anti-PD-1/L1 blockade in a variety of tumor types (Cottrell and Taube, 2018). Loss of JAK1 led to no response to anti-PD-1/PD-L1 immunotherapy (Zaretsky et al., 2016). Thus, it suggested that 1p/19q codel LGGs may not benefit from immune checkpoint blockade therapy, such as anti-PD-1/PD-L1 therapy. What is notable is that expression of CD73, a novel immune checkpoint that promotes tumor progression by suppressing anti-tumor immune response and promoting angiogenesis (Chen S. et al., 2019), was significantly higher in 1p/19q codel LGG than 1p/19q non-codel LGGs. A recent study has shown that CD73 was a specific immunotherapeutic target to improve antitumor immune responses to immune checkpoint therapy in glioblastoma multiforme (high grade glioma). Thus, our results suggest that CD73 could be a potential immunotherapeutic target for 1p/19q codel LGG patients, which needs to be confirmed by further preclinical/clinical research. In addition, it should be noted that TGF-β pathway plays a dual role in tumor progression. It promotes tumor progression in advanced stages of cancer, however, functions as a potent tumor suppressor by inducing growth inhibition and apoptosis in pre-malignant cells (Lebrun, 2012). It might partly explain that 1p/19q codel, which could impair the TGF-β pathway, is an early event in LGGs.

In conclusion, our study showed that 1p/19q codeletion plays a crucial role in regulating immune cell infiltration and expression of multiple immune Checkpoint genes in LGGs, which might be associated with tumor progression and patient survival in LGGs.

## Data Availability Statement

Publicly available datasets were analyzed in this study. This data can be found here: TCGA LGG: https://xenabrowser.net/datapages/ and CGGA: http://www.cgga.org.cn/download.jsp.

## Ethics Statement

The studies involving human participants were reviewed and approved by University of Science and Technology of China. Written informed consent for participation was not required for this study in accordance with the national legislation and the institutional requirements.

## Author Contributions

QY conceived the strategy. YJZ, QW, and YZ performed data collection. QY, LL, and YLZ performed bioinformatics analyses. QY and LL wrote the manuscript. All authors read and approved the final manuscript.

## Conflict of Interest

The authors declare that the research was conducted in the absence of any commercial or financial relationships that could be construed as a potential conflict of interest.

## Footnotes

1 https://xenabrowser.net/datapages/

2 https://www.nejm.org/doi/suppl/10.1056/NEJMoa1402121/suppl_file/nejmoa1402121_appendix_2.xlsx

3 http://www.cgga.org.cn/download.jsp

4 https://cistrome.shinyapps.io/timer

5 https://xcell.ucsf.edu

6 https://immport.org/

7 https://www.genenames.org/

## Supplementary Material

Supplementary Figure 1 Estimating the infiltrating level of stromal cells, immune cells, and tumor purity in LGG using ESTIMATE algorithm. **(A–C)** Comparison of stromal score between IDH mutant and IDH wildtype LGGs. **(D–F)** Comparison of stromal score between 1p/19q codel and 1p/19q non-codel LGGs. **(G–I)** Comparison of immune score between IDH mutant and IDH wildtype LGGs. **(J–L)** Comparison of immune score between 1p/19q codel and 1p/19q non-codel LGGs. **(M–O)** Comparison of ESTIMATE score (tumor purity) between IDH mutant and IDH wildtype LGGs. **(P–R)** Comparison of ESTIMATE score (tumor purity) between 1p/19q codel and 1p/19q non-codel LGGs. The number of patients in each subgroup was shown in Figure 1. The mean scores are indicated by red lines. *P* values are inferred from a two-sided Student’s *t*-test or Mann-Whitney test. **p* < 0.05, ***p* < 0.01, ****p* < 0.001, *****p* < 0.0001.

Supplementary Figure 2 Estimating the infiltrating level of 34 kinds of immune cells in CGGA325 LGG using xCell tool. The heatmap shows the infiltrating levels of immune cells in different subgroups of LGG patients in CGGA325 LGG. The number of patients in each subgroup was shown in Figure 1. *P* values are inferred from a two-sided Student’s *t*-test or Mann-Whitney test. **p* < 0.05, ***p* < 0.01, ****p* < 0.001, *****p* < 0.0001.

Supplementary Figure 3 Estimating the infiltrating level of 34 kinds of immune cells in CGGA693 LGG using xCell tool. The heatmap shows the infiltrating levels of immune cells in different subgroup LGG patients in CGGA693 LGG. The number of patients in each subgroup was shown in Figure 1. *P* values are inferred from a two-sided Student’s *t*-test or Mann-Whitney test. **p* < 0.05, **textitp < 0.01, ****p* < 0.001, *****p* < 0.0001.

Supplementary Figure 4 Expression of marker genes for immune cells in CGGA325 LGG. The heatmap shows the expression levels of marker genes for CD4 + T cells, effector CD8 + T cells, NK cells, B cells, T-reg cells, dendritic cells, and macrophages among different subgroup LGGs in CGGA325 LGG. *P* values are inferred from a two-sided Student’s *t*-test or Mann-Whitney test. The number of patients in each subgroup was shown in Figure 1. **p* < 0.05, ***p* < 0.01, ****p* < 0.001, *****p* < 0.0001.

Supplementary Figure 5 Expression of marker genes for immune cells in CGGA693 LGG. The heatmap shows the expression levels of marker genes for CD4 + T cells, effector CD8 + T cells, NK cells, B cells, T-reg cells, dendritic cells, and macrophages among different subgroup LGGs in CGGA693 LGG. *P* values are inferred from a two-sided Student’s *t*-test or Mann-Whitney test. The number of patients in each subgroup was shown in Figure 1. **p* < 0.05, ***p* < 0.01, ****p* < 0.001, *****p* < 0.0001.

Supplementary Figure 6 Differentially expressed immune checkpoint genes among different subgroups in CGGA325 and CGGA693 LGG. The heatmap shows the expression levels of immune-related genes on chromosome 1p/19q in different subgroups of LGG patients in CGGA325 LGG **(A)** and CGGA693 LGG **(B)**. *P* values are inferred from a two-sided Student’s *t*-test or Mann-Whitney test. The number of patients in each subgroup was shown in Figure 1. **p* < 0.05, ***p* < 0.01, ****p* < 0.001,*****p* < 0.0001.

Supplementary Figure 7 Expression of immune-related genes on chromosome 1p/19q in CGGA325 and CGGA693 LGG. Heatmap of differentially expressed immune-related genes in LGG patients in different subgroups of patients in CGGA325 LGG **(A)** and CGGA693 LGG **(B)**.

Supplementary Figure 8 The expression of CSF1, TGFB1, and JAK1 in different subgroups classified by IDH mutation and 1p/19q codel status in CGGA325 LGG and CGGA693 LGG. The mean mRNA expression was indicated by red lines. *P* values are inferred from a two-sided Student’s *t*-test or Mann-Whitney test. (**p* < 0.05, ***p* < 0.01, ****p* < 0.001, *****p* < 0.0001).

Supplementary Table 1 List of biological processes negatively associated with IDH mutation in LGG datasets.

Supplementary Table 2 List of biological processes negatively associated with IDH mutation in 1p/19q non-codeletion LGGs of all three LGG datasets.

Supplementary Table 3 List of biological processes negatively associated with 1p/19q codeletion (Codel) of all three LGG datasets.

Supplementary Table 4 List of biological processes negatively associated with 1p/19q codeletion in muIDH LGGs of all three LGG datasets.

Supplementary Table 5 List of immune-related genes mapped on chromosome 1p and 19q.

## Abbreviations

19q: q-arm of chromosome 19
1p: p-arm of chromosome 1
BP: biological processes
CGGA: Chinese Glioma Genome Atlas
FDR: false discovery rate
GBM: Glioblastoma
GEM: genetically engineered mice
GSEA: Gene Set Enrichment Analysis
ICB: immune checkpoint blockade
IDH: isocitrate dehydrogenase
LGG: low grade glioma
MSigDB: molecular signatures database
NES: normalized enrichment score
TAM: tumor associated macrophage
TCGA: The cancer genome atlas
TIICs: tumor-infiltrating immune cells
TIMER: tumor immune estimation resource
WHO: World Health Organization.
