# Supplementary figures and images for: Effects of 1p/19q Codeletion on Immune Phenotype in Low Grade Glioma
Source: Front Cell Neurosci. 2021 Jul 16;15:704344. doi: 10.3389/fncel.2021.704344 (PMC8322528; doi:10.3389/fncel.2021.704344)

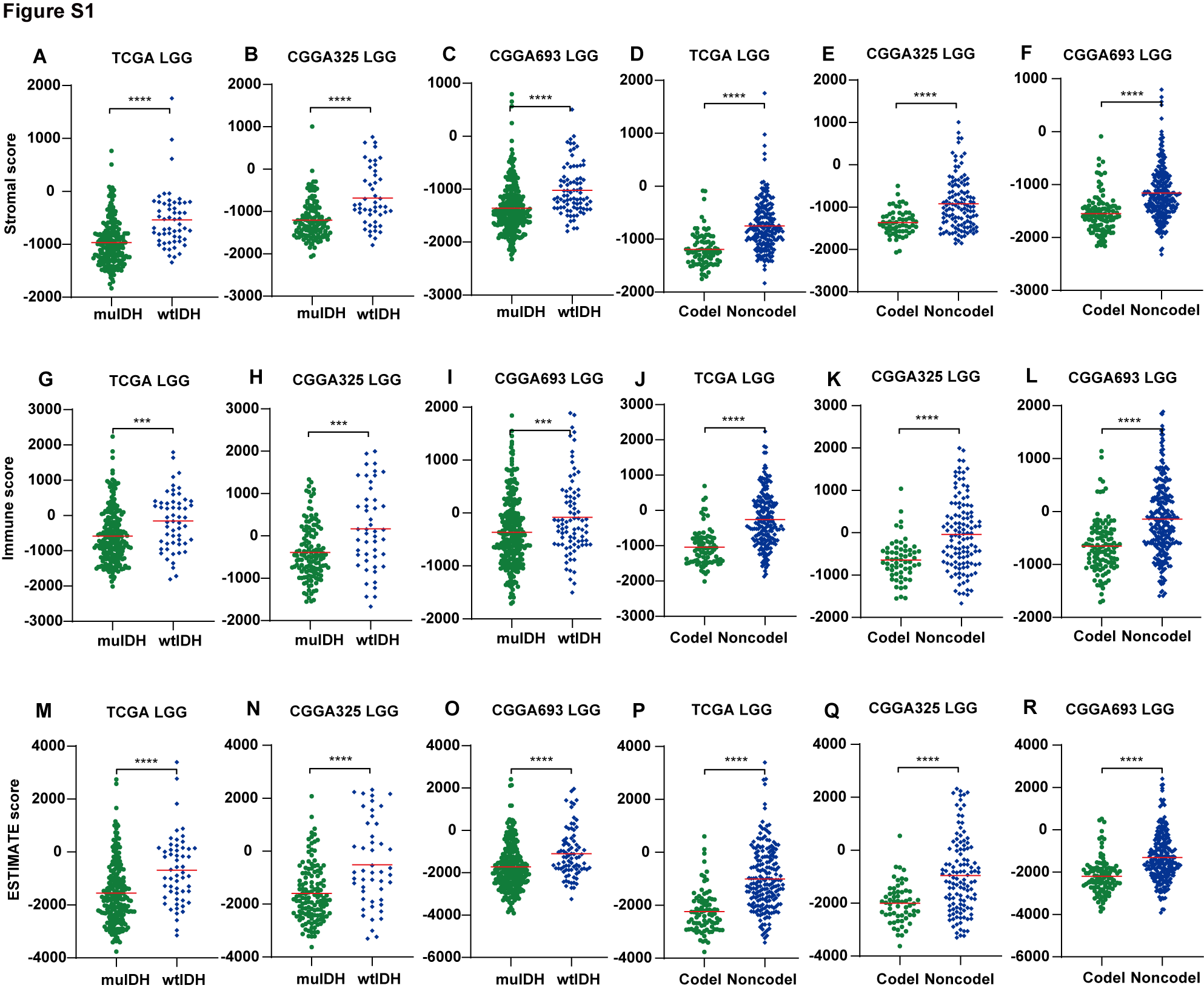

Supplement: Supplementary Figure 1 — Estimating the infiltrating level of stromal cells, immune cells, and tumor purity in LGG using ESTIMATE algorithm. (A–C) Comparison of stromal score between IDH mutant and IDH wildtype LGGs. (D–F) Comparison of stromal score between 1p/19q codel and 1p/19q non-codel LGGs. (G–I) Comparison of immune score between IDH mutant and IDH wildtype LGGs. (J–L) Comparison of immune score between 1p/19q codel and 1p/19q non-codel LGGs. (M–O) Comparison of ESTIMATE score (tumor purity) between IDH mutant and IDH wildtype LGGs. (P–R) Comparison of ESTIMATE score (tumor purity) between 1p/19q codel and 1p/19q non-codel LGGs. The number of patients in each subgroup was shown in Figure 1. The mean scores are indicated by red lines. P values are inferred from a two-sided Student’s t-test or Mann-Whitney test. *p < 0.05, **p < 0.01, ***p < 0.001, ****p < 0.0001. [file Image_1.TIF]

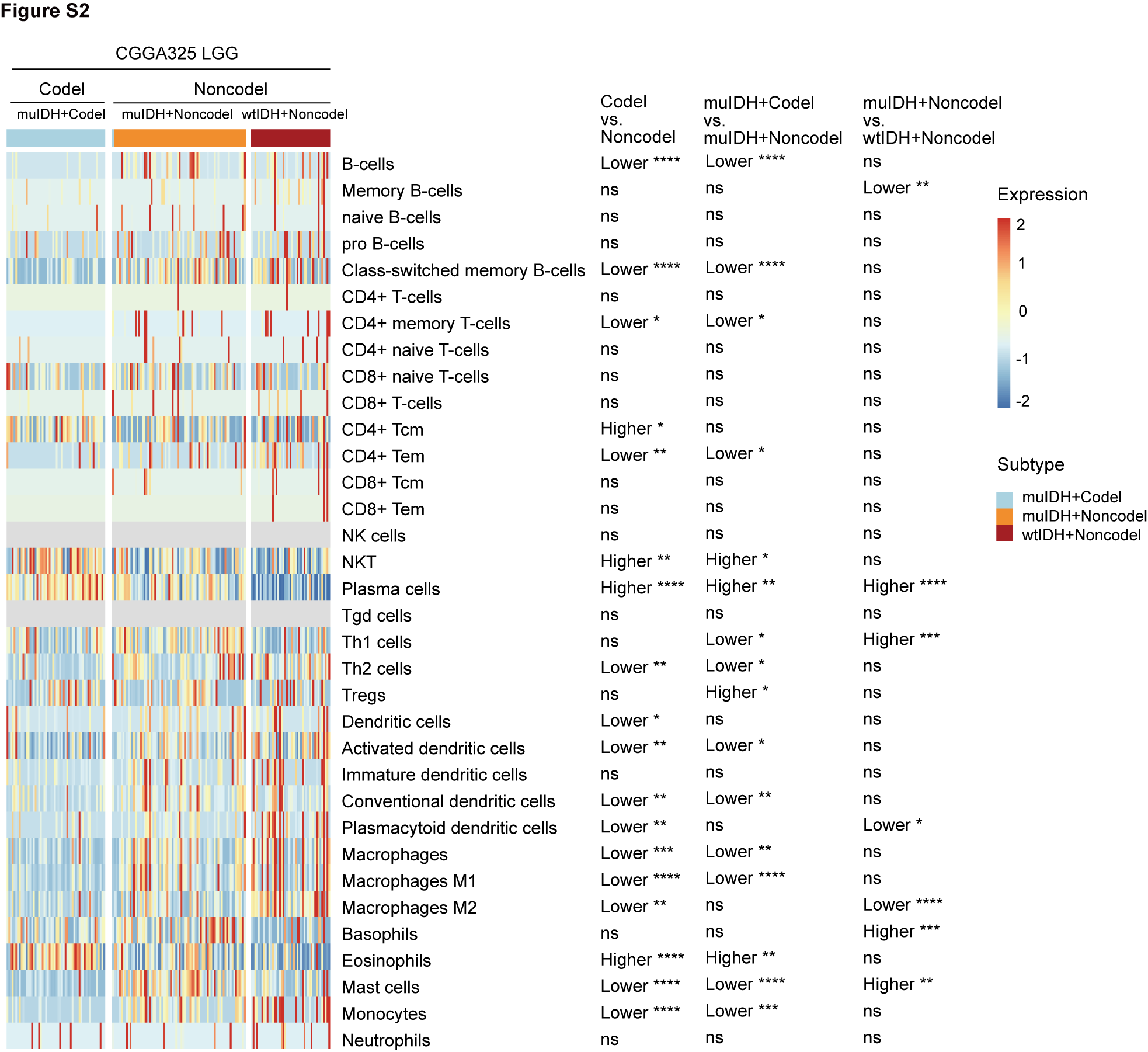

Supplement: Supplementary Figure 2 — Estimating the infiltrating level of 34 kinds of immune cells in CGGA325 LGG using xCell tool. The heatmap shows the infiltrating levels of immune cells in different subgroups of LGG patients in CGGA325 LGG. The number of patients in each subgroup was shown in Figure 1. P values are inferred from a two-sided Student’s t-test or Mann-Whitney test. *p < 0.05, **p < 0.01, ***p < 0.001, ****p < 0.0001. [file Image_2.TIF]

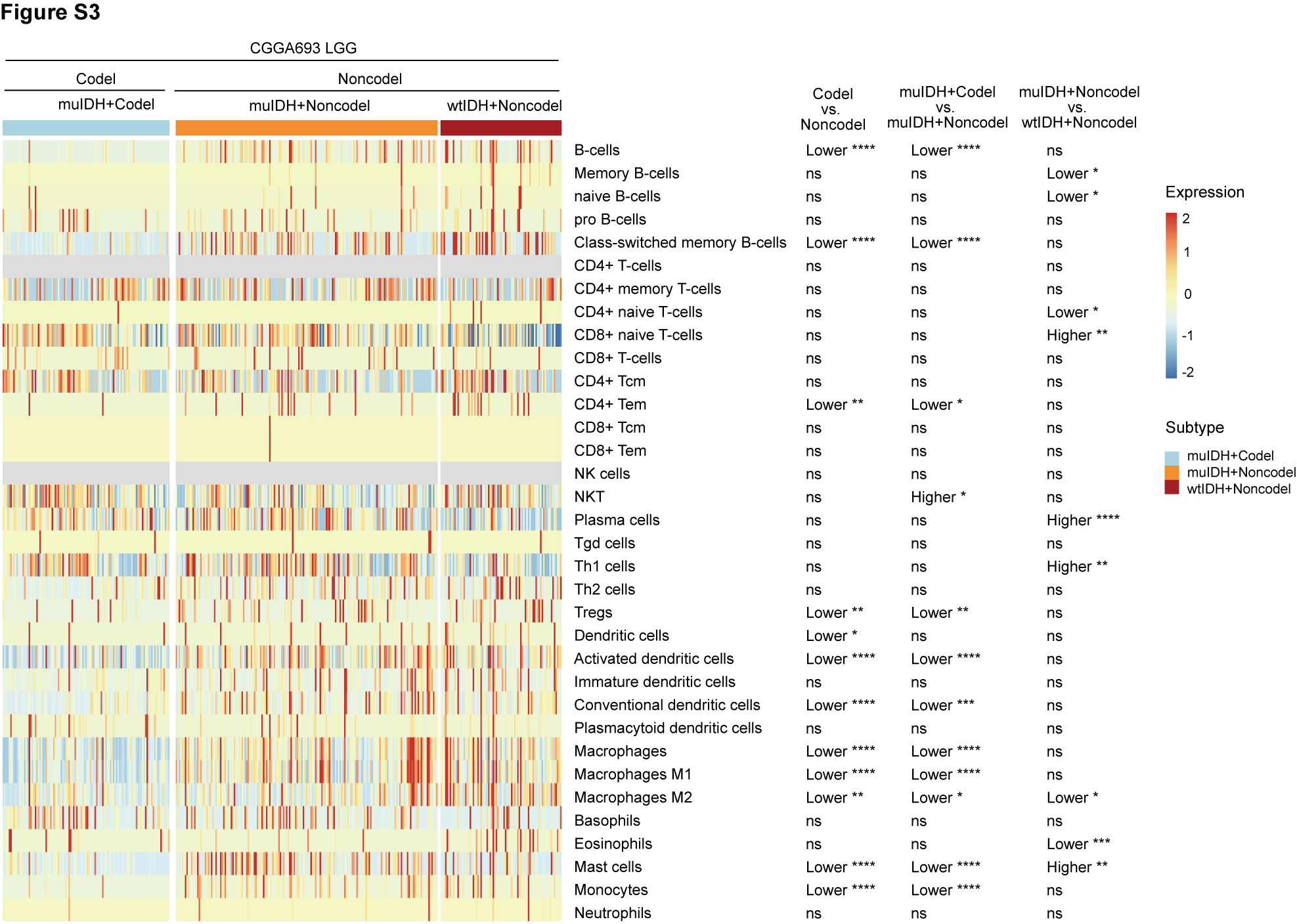

Supplement: Supplementary Figure 3 — Estimating the infiltrating level of 34 kinds of immune cells in CGGA693 LGG using xCell tool. The heatmap shows the infiltrating levels of immune cells in different subgroup LGG patients in CGGA693 LGG. The number of patients in each subgroup was shown in Figure 1. P values are inferred from a two-sided Student’s t-test or Mann-Whitney test. *p < 0.05, **textitp < 0.01, ***p < 0.001, ****p < 0.0001. [file Image_3.TIF]

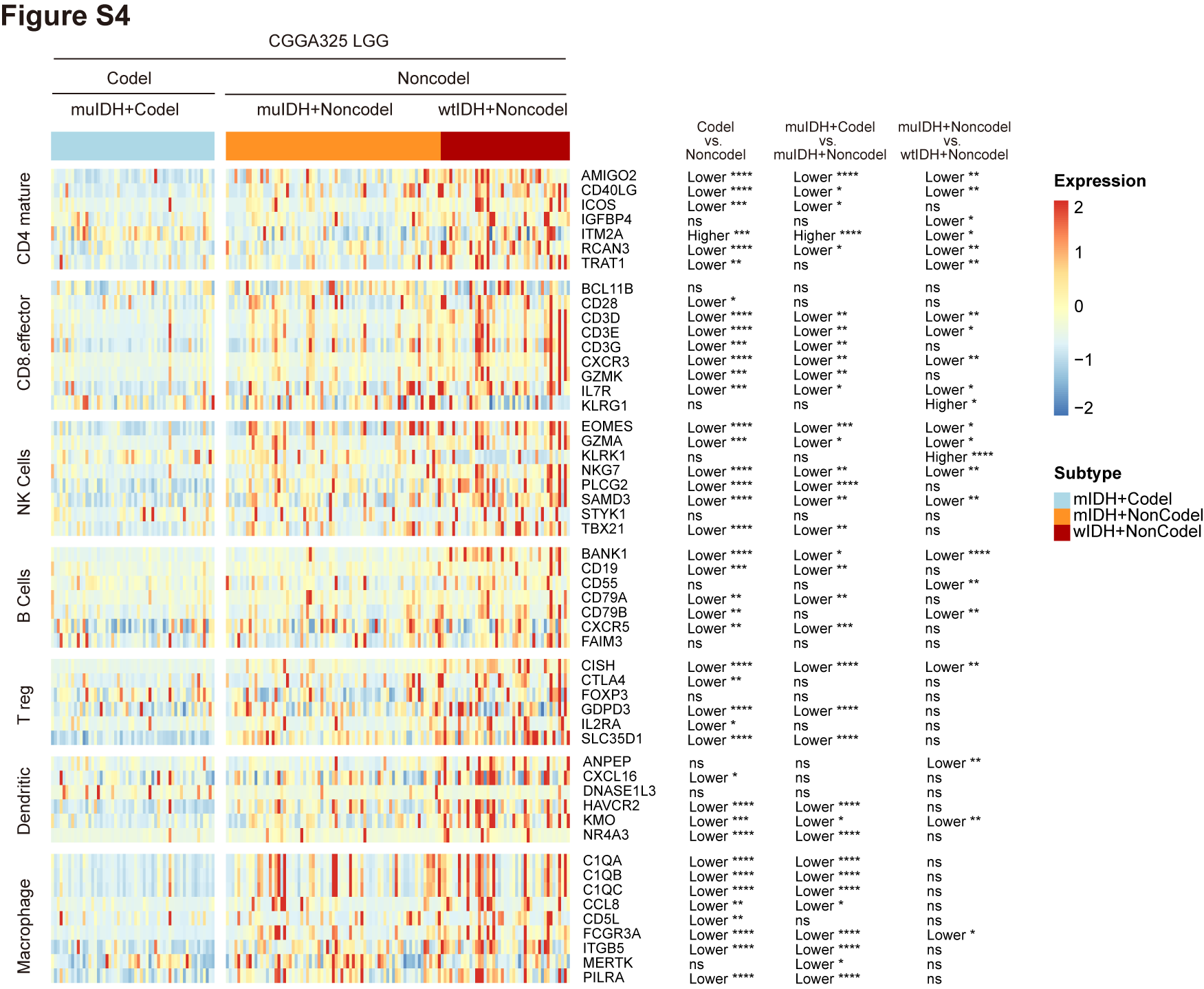

Supplement: Supplementary Figure 4 — Expression of marker genes for immune cells in CGGA325 LGG. The heatmap shows the expression levels of marker genes for CD4 + T cells, effector CD8 + T cells, NK cells, B cells, T-reg cells, dendritic cells, and macrophages among different subgroup LGGs in CGGA325 LGG. P values are inferred from a two-sided Student’s t-test or Mann-Whitney test. The number of patients in each subgroup was shown in Figure 1. *p < 0.05, **p < 0.01, ***p < 0.001, ****p < 0.0001. [file Image_4.TIF]

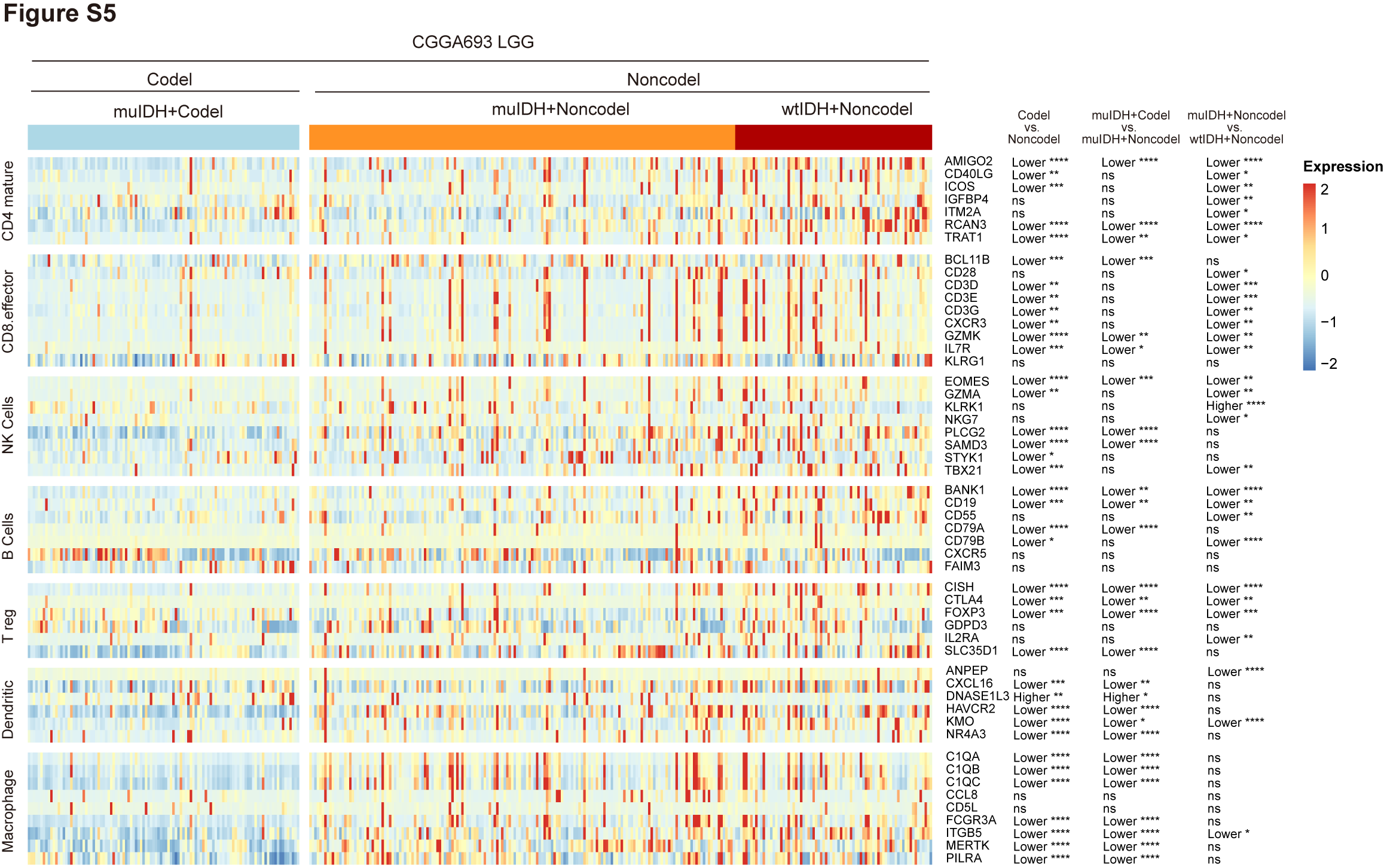

Supplement: Supplementary Figure 5 — Expression of marker genes for immune cells in CGGA693 LGG. The heatmap shows the expression levels of marker genes for CD4 + T cells, effector CD8 + T cells, NK cells, B cells, T-reg cells, dendritic cells, and macrophages among different subgroup LGGs in CGGA693 LGG. P values are inferred from a two-sided Student’s t-test or Mann-Whitney test. The number of patients in each subgroup was shown in Figure 1. *p < 0.05, **p < 0.01, ***p < 0.001, ****p < 0.0001. [file Image_5.TIF]

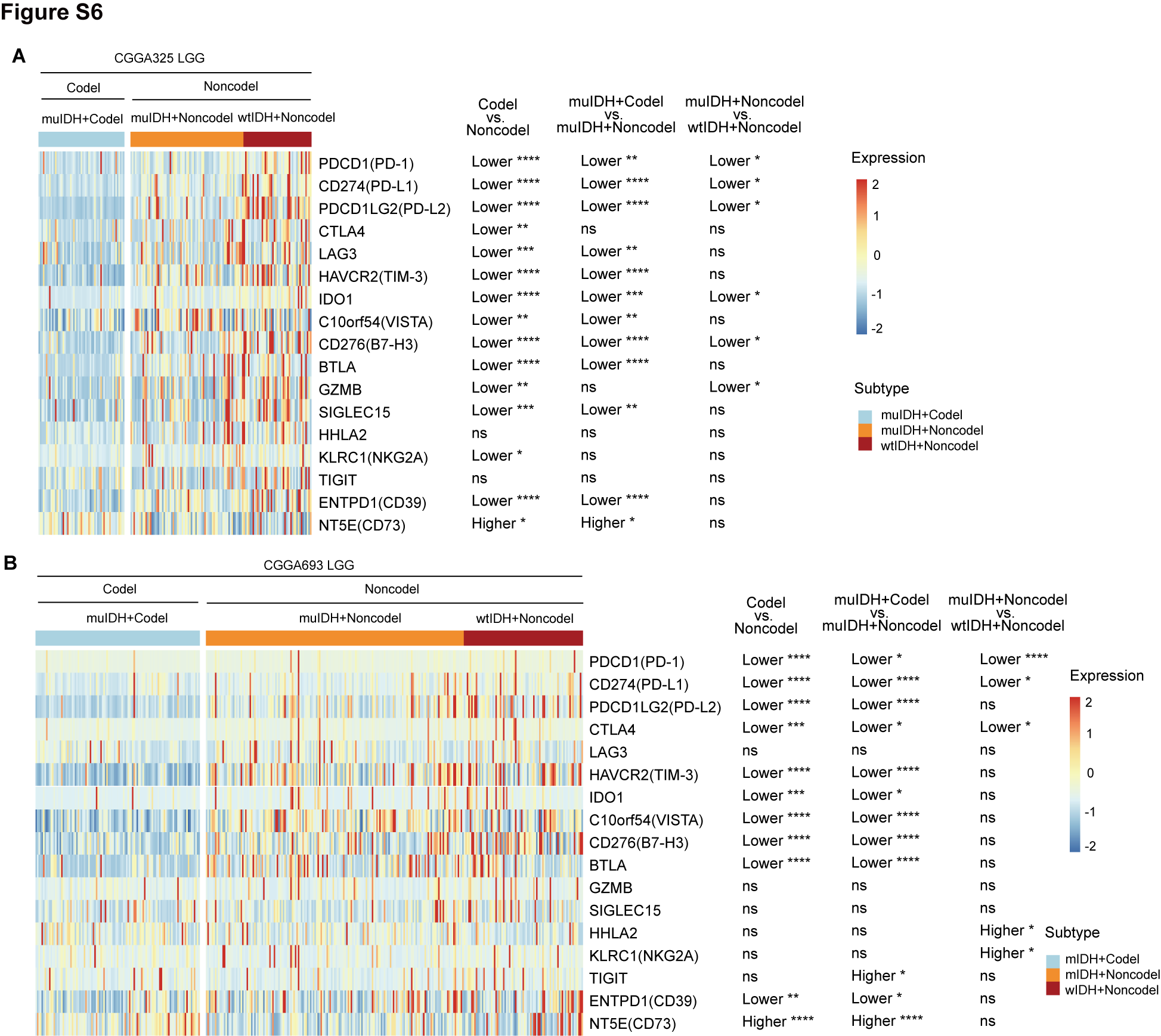

Supplement: Supplementary Figure 6 — Differentially expressed immune checkpoint genes among different subgroups in CGGA325 and CGGA693 LGG. The heatmap shows the expression levels of immune-related genes on chromosome 1p/19q in different subgroups of LGG patients in CGGA325 LGG (A) and CGGA693 LGG (B). P values are inferred from a two-sided Student’s t-test or Mann-Whitney test. The number of patients in each subgroup was shown in Figure 1. *p < 0.05, **p < 0.01, ***p < 0.001,****p < 0.0001. [file Image_6.TIF]

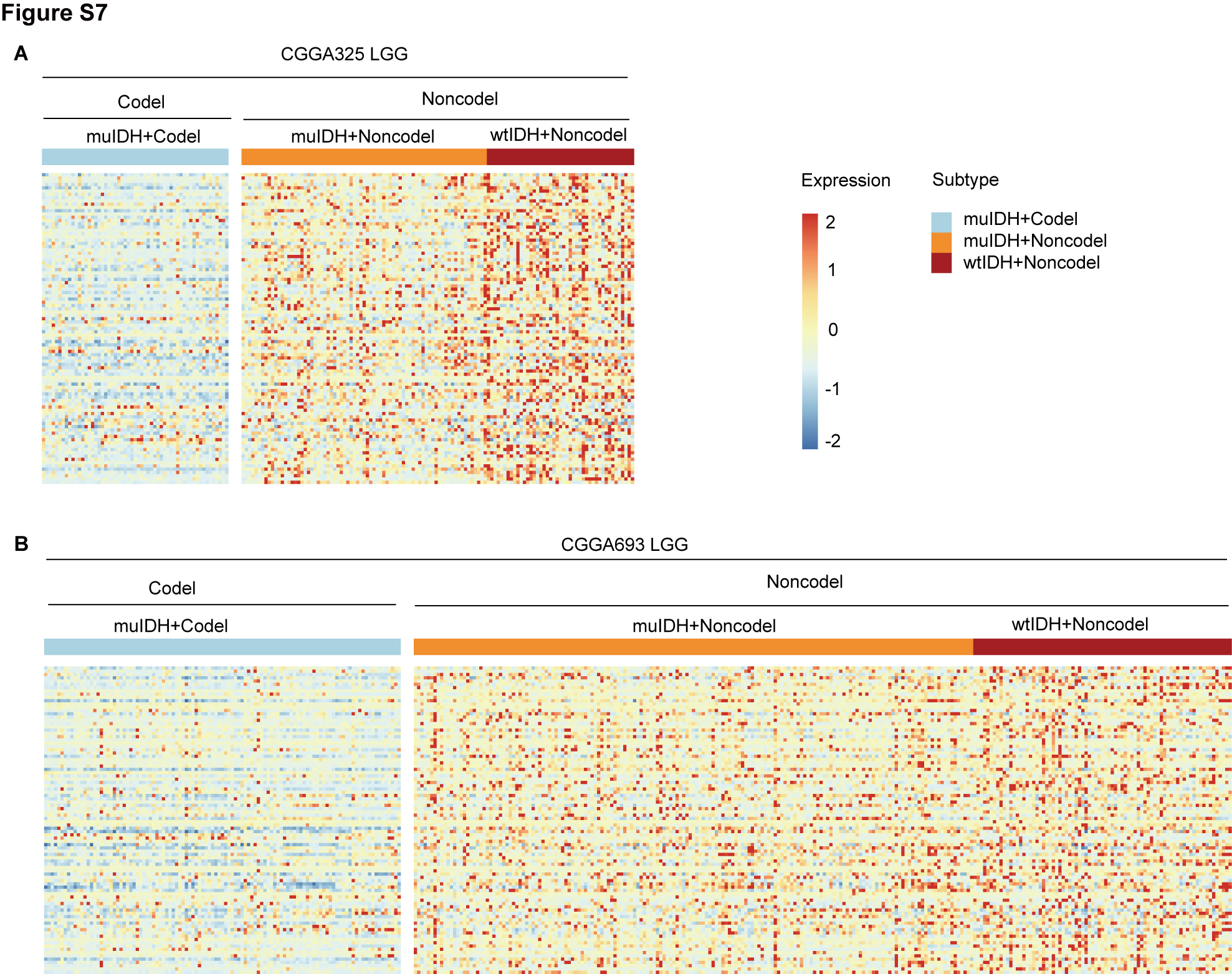

Supplement: Supplementary Figure 7 — Expression of immune-related genes on chromosome 1p/19q in CGGA325 and CGGA693 LGG. Heatmap of differentially expressed immune-related genes in LGG patients in different subgroups of patients in CGGA325 LGG (A) and CGGA693 LGG (B). [file Image_7.TIF]

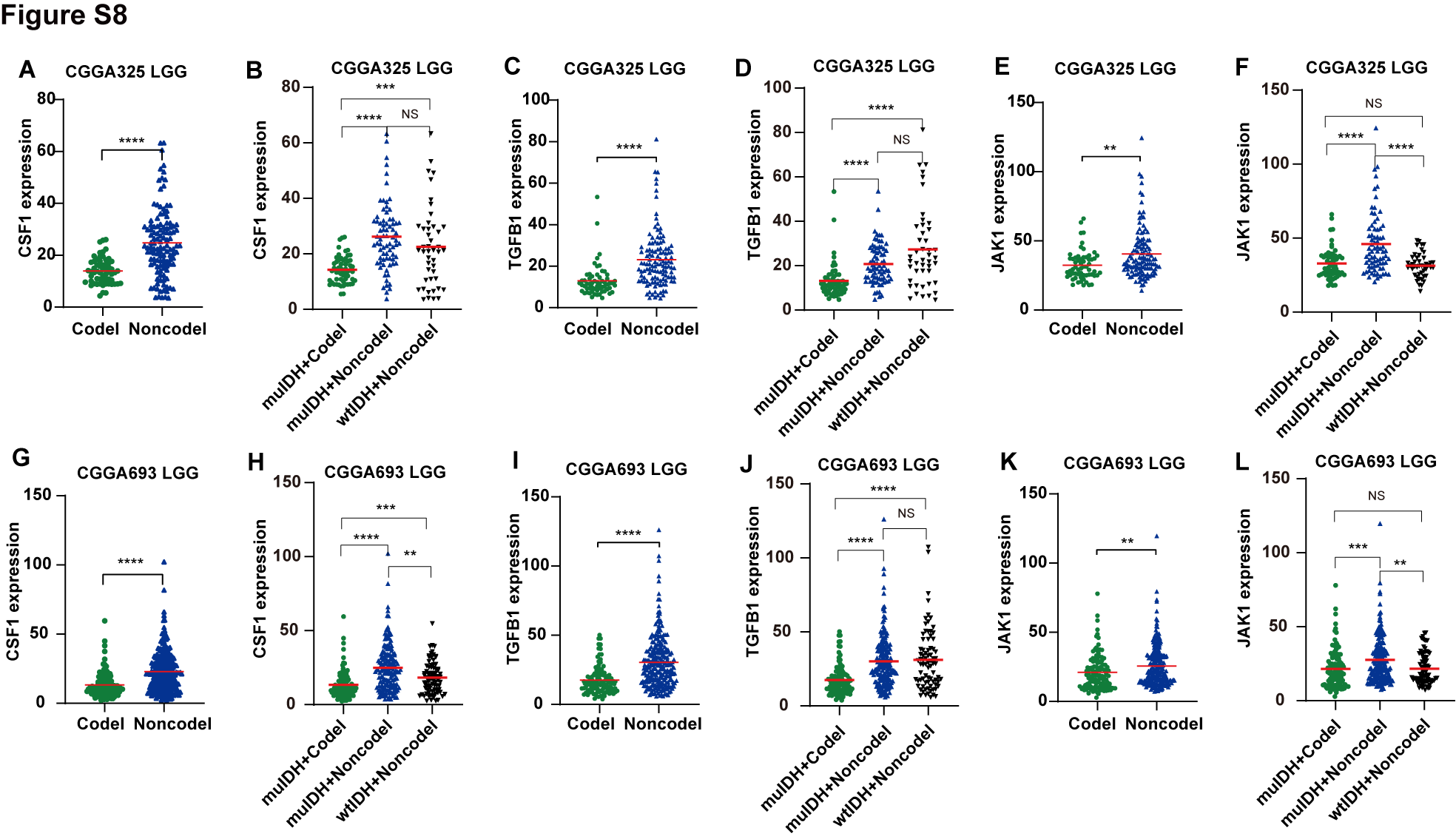

Supplement: Supplementary Figure 8 — The expression of CSF1, TGFB1, and JAK1 in different subgroups classified by IDH mutation and 1p/19q codel status in CGGA325 LGG and CGGA693 LGG. The mean mRNA expression was indicated by red lines. P values are inferred from a two-sided Student’s t-test or Mann-Whitney test. (*p < 0.05, **p < 0.01, ***p < 0.001, ****p < 0.0001). [file Image_8.TIF]
